# Downregulated PRNP Facilitates Cell Proliferation and Invasion and Has Effect on the Immune Regulation in Ovarian Cancer

**DOI:** 10.1155/2022/3205040

**Published:** 2022-09-29

**Authors:** Kuan Hu, Xiaofang Zhang, Lei Zhou, Juanni Li

**Affiliations:** ^1^Department of Hepatobiliary Surgery, Xiangya Hospital, Central South University, Changsha, 410008 Hunan, China; ^2^Departments of Burn and Plastic, Ningxiang People's Hospital, Hunan University of Chinese Medicine, Changsha, 410600 Hunan, China; ^3^Department of Anesthesiology, Third Xiangya Hospital of Central South University, Changsha, 410008 Hunan, China; ^4^Department of Pathology, Xiangya Hospital, Central South University, Changsha, 410008 Hunan, China; ^5^National Clinical Research Center for Geriatric Disorders, Xiangya Hospital, Central South University, Changsha, 410008 Hunan, China

## Abstract

**Background:**

Ovarian cancer (OC) seriously threatens women's life. Ferroptosis plays an essential role in the initiation and development of OC. However, more molecular targets and mechanisms for ferroptosis in OC remain to be further elucidated.

**Methods:**

Several OC datasets were integrated in this study and three candidate genes including PRNP were further screened out as the ferroptosis-related gene which was differentially expressed in OC. Then, comprehensive evaluations concerning gene expression, clinical implication, in vitro validation of expression and functional experiments, prediction of downstream molecules and related signal pathways, and immune-modulating function were performed.

**Results:**

PRNP was the only downregulated ferroptosis-related gene with prognostic value for OC patients. The decreased mRNA and protein expression was verified in OC tissues and cell lines. PRNP was significantly correlated with cancer stages, primary therapy outcomes, and age in OC patients. Moreover, we found that overexpression of PRNP inhibited the proliferation, migration, and invasion ability of OC cells through in vitro experiments. PRNP was enriched to the Ras signaling pathway. PRNP expression was positively correlated with the infiltration of immune cells, such as mast cells, T effector memory cells, plasmacytoid DC cells, NK cells, and eosinophils. In addition, the association of PRNP with other immune signatures was also found.

**Conclusion:**

This study demonstrated for the first time showed that ferroptosis-related gene PRNP exerted a tumor suppressive role in OC and the aberrant expression and function of PRNP making it a potential novel biomarker for OC diagnosis, prognosis, and response to immunotherapies.

## 1. Introduction

Ovarian cancer (OC) is one of the most common cancers that happened in the reproductive organs of women, with the incidence ranked third and mortality ranked first among gynecological malignancies, thereby seriously threatening women's life [1, 2]. OC is characterized by its latent initiation and high grade of malignancy, which results in the advanced stage of OC patients when they are first diagnosed and poor survival [3, 4]. Therefore, investigations to unveil the uncleared mechanisms underneath the initiation of OC are constantly urgent.

Ferroptosis is a novel form of programmed cell death based on iron metabolism and oxidation-reduction reactions [5]. Nowadays, ferroptosis has become a hotspot of oncological research and a promising mechanism to improve diagnostic effectiveness and survival for patients with cancers [6, 7]. Some researches were exploring the role of ferroptosis in OC. For instance, PARP was reported to inhibit ferroptosis through SC7A11 and other ferroptosis inducers in BRCA-proficient OC [8]. Moreover, OC cells with platinum tolerance were proved to be of high susceptibility to ferroptosis when biomarker Frizzed-7 was overexpressed. Stemness features and altered glutathione metabolism via GPX4 may account for the mechanism behind [9]. However, more molecular targets and mechanisms for ferroptosis in OC remain to be further elucidated.

Here, three public datasets were recruited in this study for further analysis, and ferroptosis-related genes that were differentially expressed in OC were filtered and identified as candidate genes that were involved in the initiation and development of OC. PRNP draw our great attention to these candidate genes when comprehensive evaluations concerning gene expression, clinical implication, in vitro validation of functional experiments, prediction of downstream molecules and related signal pathways, and immune-modulating function were performed. PRNP (prion protein) is a compact gene located at human chromosome 20p, with only an open reading frame in structure and containing no intron [10]. PRNP gene encodes cellular prion protein, a high conserved glycoprotein on cell membrane with various functions ranging from antiapoptosis, cellular signaling regulation, and autophagy, to cell migration [11–14]. Aberrant expression of PRNP has been observed in many types of cancer, including cancers of the digestive system melanoma and oral squamous cell carcinoma [15–19]. However, there has been no study exploring the role of PRNP in OC, especially from the perspective of ferroptosis. This study is aimed at filling this vacancy, as well as offer a novel thought for further investigating the potential mechanism of PRNP-induced ferroptosis in regulating OC.

## 2. Materials and Methods

### 2.1. The Data Acquirement and Reanalysis

In brief, two datasets containing information of ovarian cancer tissues and normal ovarian tissues were downloaded from the Gene Expression Omnibus (GEO) database, along with the downloading of ferroptosis-related genes in a dataset from the MalaCards database (https://www.malacards.org/) [20, 21]. These three datasets were further used for filtering the overlapping genes, namely, codifferentially expressed and ferroptosis-related genes in ovarian cancer via Venn diagram.

We further evaluated the prognostic role of three candidate genes in OC with the help of Kaplan-Meier plotter (http://kmplot.com/analysis/), an online public resource retrieving gene expressions and survival data from public cancer databases such as TCGA for prognostic analysis [22]. GEPIA2.0 (http://gepia.cancer-pku.cn/) and UALCAN databases (http://ualcan.path.uab.edu/index.html) were employed to show the differential mRNA and protein expression of PRNP in OC. *p* value less than 0.05 was presumed to have statistical significance. The expression difference of UALCAN among each stage of OC was performed by using UALCAN database [23–25]. We used TCGA-OC to generate the coexpression gene network of PRNP in OC by using Xiantao tool (https://www.xiantao.love) [26, 27]. Based on this gene network, the Gene Ontology (GO) analysis and the Kyoto Encyclopedia of Genes and Genomes (KEGG) analysis were conducted. The correlation analysis between PRNP and immune cell infiltration was performed by using TCGA-OC database through Xiantao tool, a comprehensive online portal focused on tumor and immune system interaction. The protein expression of the markers of different immune cells was obtained from The Human Protein Atlas database (https://www.proteinatlas.org) [28].

### 2.2. Cell Culture

The ovarian cell lines SKOV3 and HO8910 were cultured as previously described [29].

### 2.3. Overexpression of PRNP in OC Cell Lines

PRNP overexpression plasmid pcDNA3.1-PRNP-flag was purchased from HonorGene (HG-HO000311). PRNP PLASMID was transfected into SKOV3 and HO8910 cell lines with the participation of lipofectamine 2000 for 24 h. Western blots were performed to validate the efficacy of transfection.

### 2.4. Scratch Wound-Healing Assay, Transwell Assay, and CCK8 Assay

Scratch wound-healing assay was performed when ovarian cancer cells reached about 95% confluence in culture dishes. Briefly, after 24 h of starvation through the serum-free medium, scratches were made in each culture dish by using a sterile pipette tip (10 *μ*l), and two times of mild washing were followed to remove the debris of cells. Images were taken at the time points of 0 h, 12 h, and 24 h, respectively, using a microscope. Transwell assay and CCK8 assay were performed according to the protocols we previously described [30].

### 2.5. qPCR and Western Blot

qPCR and Western blot were performed according to the protocols we previously described [30]. The PNRP primer was: F 5′-AGAGGCCCAGGTCACTCC-3′, R 5′-GAGCTTCTCCTCTCCTCACG-3′. The PRNP antibody (ab52604) and GAPDH antibody (KM9002) were diluted as 1 : 1000. The procedure of RNA and protein abstraction for ovarian cancer cell lines was performed under the direction of the manufacturer's instrument.

### 2.6. Statistical Analysis

SPSS 20.0 software (SPSS, Chicago, IL) was used for statistical analysis in this study. Unpaired continuous data were analyzed by Student's *t*-test. The prognostic role of PRNP expression was evaluated through the Kaplan-Meier method and Log-rank test. A cutoff of *p* value equal to 0.05 was set to have statistical significance. Statistical analysis from online databases was also included, such as Kaplan-Meier Plotter (http://kmplot.com/) and UALCAN.

## 3. Results

### 3.1. Identification of Differentially Expressed Genes

Through screening the differentially expressed genes (DEGs) between ovarian cancers and normal ovary, we analyzed two publicly ovarian cancer GEO datasets, and the criteria for screening were set as *p* < 0.05 and ∣log2 FC | >1. The results showed that 2332 upregulated genes and 1003 downregulated genes were identified in GSE12470 (Supplementary Table S1), 726 upregulated genes, and 1296 downregulated genes were identified in GSE26712 (Supplementary Table S2).

**Figure 1:**
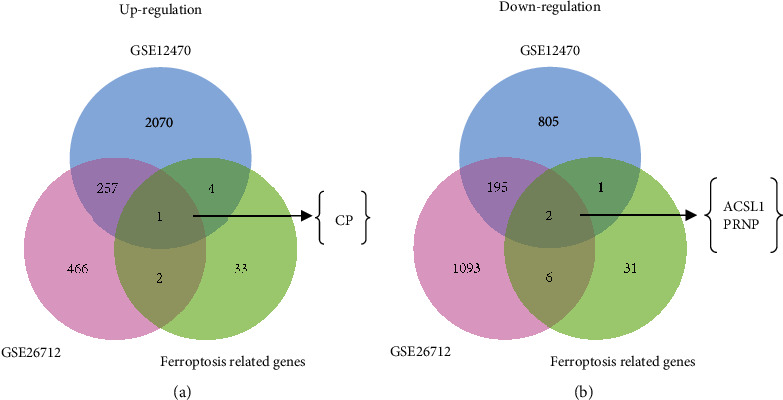
Venn diagrams to ferroptosis-related genes that were differentially expressed in ovarian cancer from microarray datasets. (a) Coincreased expression genes from public ovarian cancer datasets (GSE12470 and GSE26712) were filtered together with ferroptosis-related genes to identify the candidate gene CP. (b) Codecreased expression genes from public ovarian cancer datasets (GSE12470 and GSE26712) were filtered together with ferroptosis-related genes to identify the candidate genes ACSL1 and PRNP.

Increasing evidence showed that ferroptosis played an important role in the development of ovarian cancer. Thus, we further explore the potential role of ferroptosis on the development of OC. The overlapping co-DEGs between the ferroptosis-related gene dataset and two ovarian cancer datasets were analyzed through Venn analysis, and the results exhibited that one upregulated ferroptosis-related gene, CP, and two downregulated ferroptosis-related genes, ACSL1 and PRNP, were found, respectively (Figure 1, Supplementary Table S3). These ferroptosis-related genes were hypothesized to be involved in the development of ovarian cancer.

### 3.2. PRNP Exhibits the Good Prognostic Value in OC

**Figure 2:**
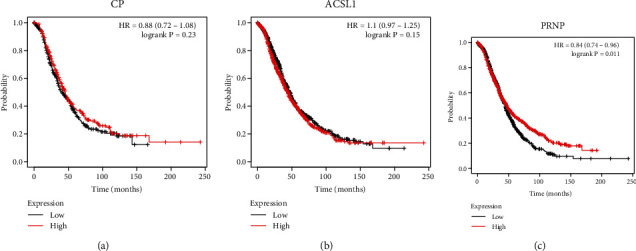
Survival analysis of the three differentially expressed ferroptosis-associated genes in patients with OC (Kaplan-Meier Plotter). (a) The association between CP expression and overall survival. (b) The association between ACSL1 expression and overall survival. (c) The association between PRNP expression and overall survival.

Next, we further explored the impact of these three ferroptosis-related genes on the prognosis of ovarian cancer patients. As shown in Figure 2, only PRNP showed that its high expression was associated with longer overall survival (OS) of ovarian cancer patients, and the other two genes, CP and ACSL1, showed no association with the prognosis of OC. These findings suggested that this ferroptosis-related gene, PRNP, had potential roles in the prognosis of OC and was selected for further research (Figure 2).

### 3.3. Decreased PRNP Expression in OC and Its Association with Clinicopathologic Factors

**Figure 3:**
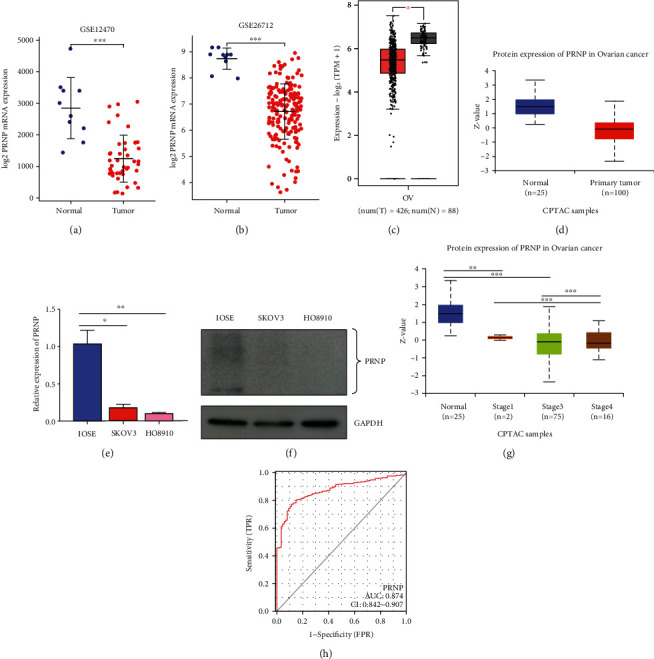
Analysis of PRNP expression in OC patients and cell lines. (a and b) The mRNA expression of PRNP in OC datasets GSE12470 and GSE26712. (c) The mRNA expression of PRNP in OC tissues returned from GEPIA 2.0 database. (d) The protein expression of PRNP in OC by using UALCAN database. (e and f) The mRNA and protein level of PRNP in OC cell lines. (g) The correlation of PRNP with cancer stages for patients with OC. (h) Evaluation of the diagnostic role of PRNP in OC. ^∗^*p* < 0.05, ^∗∗^*p* < 0.01, ^∗∗∗^*p* < 0.00.

To further validate the expression level of PRNP in ovarian cancer, we explored the transcriptional level of PRNP in two independent public ovarian cancer GEO datasets and found that compared to the normal ovarian tissues, the mRNA levels of PRNP were obviously decreased in ovarian cancer tissues (Figure 3(a) and 3(b)). We also obtained a consistent result through GEPIA2 analysis (Figure 3(c)). In addition, we found that the protein expression level of PRNP was significantly downregulated in ovarian cancer tissues based on UALCAN database (Figure 3(d)). Moreover, through in vitro experiments, we further validated that the transcriptional and protein expression levels of PRNP were significantly decreased in ovarian cancer cells SKOV3 and HO8910 compared with the normal ovarian cells IOSE (Figure 3(e) and 3(f)).

Furthermore, we explored the correlation between the PRNP expression and clinicopathological factors of ovarian cancer patients. The results showed that PRNP transcriptional expression was related to primary therapy outcomes and age based on the logistic regression analysis (Supplementary Table S4). Additionally, we also found that PRNP protein expression was associated with cancer stages of OC (Figure 3(g)). Moreover, we further applied the TCGA-OC dataset to explore the potential diagnostic value of PRNP expression in OC, and the results revealed that the AUC of PRNP was 0.874, suggesting that PRNP was a potential diagnostic biomarker for OV patients (Figure 3(h)).

### 3.4. PRNP Inhibits the Proliferation and Invasion Ability of OC Cells

**Figure 4:**
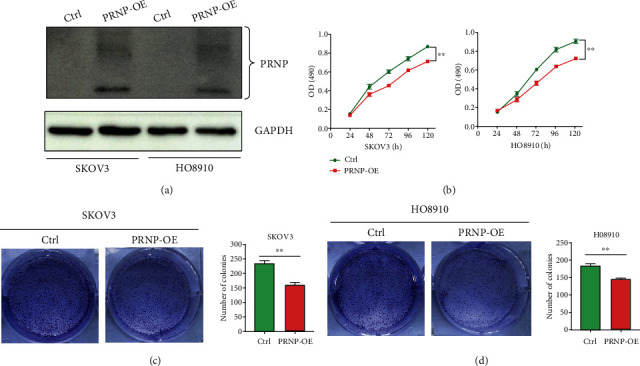
Overexpressed PRNP inhibited the proliferation of OC cell lines. (a) The expression of PRNP after PRNP overexpression plasmid treatment in two OC cell lines. (b) CCK8 assay when PRNP was overexpressed in two OC cell lines. (c and d) Colony formation assay when PRNP was overexpressed in two OC cell lines. OE: overexpression; ^∗∗^*p* < 0.01.

**Figure 5:**
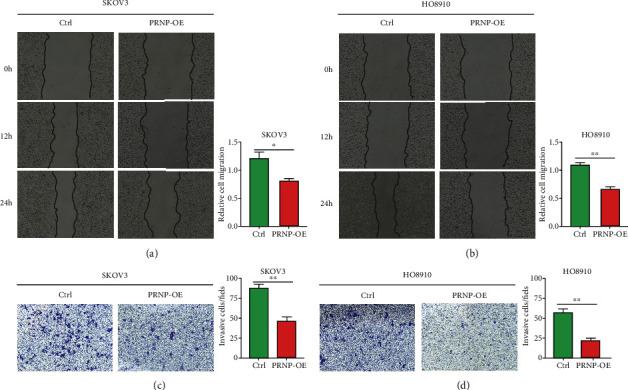
Overexpressed PRNP inhibited the migration and invasion of OC cell lines. (a and b) Scratch wound-healing assay in two OC cell lines with PRNP overexpression. (c and d) Transwell assay in two OC cell lines with PRNP overexpression. OE: overexpression; ^∗^*p* < 0.05, ^∗∗^*p* < 0.01.

Next, we further explore the role of PRNP in proliferation, migration, and invasion ability of ovarian cancer cells. First, we overexpressed the expression of PRNP in two ovarian cancer cell lines SKOV3 and HO8910. Compared to the control group, the expression level of PRNP was significantly overexpressed in the treatment group (Figure 4(a)). Then, we conducted the CCK8 assay, and the results showed that PRNP overexpression obviously decreased the proliferation ability of two ovarian cell lines (Figure 4(b)). Meanwhile, the colony formation assay also exhibited the consistence results that PRNP overexpression inhibited cell proliferation (Figure 4(c) and 4(d)). Moreover, we further investigate whether PRNP overexpression affected cell migration and invasion in OC. Through wound-healing assay and transwell assay, we observed that overexpression of PRNP could significantly inhibit the migration and invasion ability of OC cells (Figure 5(a)–5(d)). These findings revealed that PRNP could suppress some malignant biological behaviors of ovarian cancer cells and may execute its inhibitory function in the progression of ovarian cancer.

### 3.5. Biological Function and Pathways Analysis of PRNP in OC

**Figure 6:**
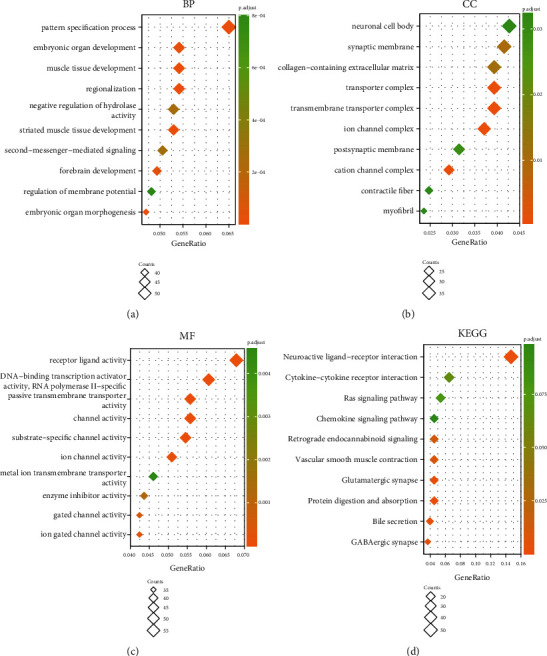
Functional enrichment analysis of PRNP in ovarian cancer. (a–c) GO (BP, CC, and MF) enrichment analysis of PRNP in ovarian cancer (Xiantao). (d) KEGG enrichment analysis of PRNP in ovarian cancer. BP: biological process; CC: cellular components; MF: molecular function.

We further analyzed the biological function and pathways of PRNP in ovarian cancer. The genes coexpressed with PRNP were analyzed, and the screening criteria were *p*value < 0.05 and ∣ logFC  | >0.6. As shown in Figure 6(a), Gene Ontology (GO) term annotation for biological process (BP) exhibited that pattern specification process, embryonic organ development, muscle tissue development, and regionalization were enriched. The cellular component (CC) analysis exhibited that these genes coexpressed with PRNP were mainly enriched in the neuronal cell body, synaptic membrane, collagen-containing extracellular matrix, and transporter complex (Figure 6(b)). GO term annotation for molecular function (MF) showed that receptor ligand activity, DNA-binding transcription activator activity, RNA polymerase II-specific, and passive transmembrane transporter activity were mainly enriched (Figure 6(c)). KEGG pathway analysis revealed that these most enriched pathways were neuroactive ligand-receptor interaction, cytokine-cytokine receptor interaction, Ras signaling pathway, and chemokine signaling pathway (Figure 6(d)).

### 3.6. Correlations between PRNP Expression and Immune Infiltrations in OC

**Figure 7:**
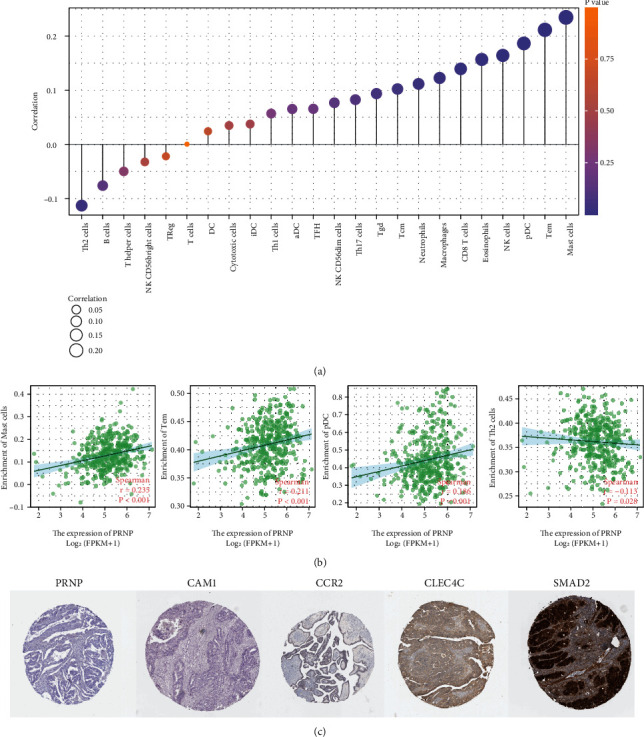
The correlation of PRNP with immune cell infiltration in OC. (a) The correlation of PRNP with each infiltrated immune cell (Xiantao). (b) The top three immune cells with the highest correlation and the one with the lowest (Xiantao). (c) Immunohistochemistry images showed the protein levels of PRNP and CAM1, CCR2, CLEC4C, and SMAD in OC (The Human Protein Atlas database).

Increasing findings reported that immune infiltration plays a critical role in tumor development and progression [31, 32]. Next, we investigated whether PRNP expression was correlated with immune infiltration in OC. The correlation between PRNP expression and the infiltrating of twenty-four immune cells was analyzed in the TCGA-OC cohort utilizing ssGSEA with the Spearman correlation (Figure 7(a)). The results exhibited that PRNP expression was positively correlated with the infiltration of some immune cells, such as mast cells (Spearman *r* = 0.235), T effector memory (Tem) cells (Spearman *r* = 0.211), plasmacytoid DC (pDC) cells (Spearman *r* = 0.186), NK cells (Spearman *r* = 0.164), and eosinophils (Spearman *r* = 0.157) and was negatively correlated with the infiltration of Th2 cells (Spearman *r* = −0.113) (Figure 7(b)). Moreover, we further explored the relationship between the expression of PRNP and the expression of gene markers of these aforementioned immune cells in ovarian cancer tissues. The staining levels of protein expression are classified as follows: not detected, low, medium, and high. As shown in Figure 7(c), we discovered that the staining of PRNP, CAM1 (a marker of mast cell) and CCR2 (a marker of Tem cell) was not detected, and the staining of CLEC4C (a marker of pDC cell) was low, however, the staining of SMAD2 (a marker of Th2 cells) was high. These findings suggested that PRNP was potentially involved in tumor immune cell infiltration in OC.

To better understand the role of PRNP in immune regulation in OC, we further analyzed the association between PRNP expression and other immune signatures. As shown in Supplementary Figure S1, the correlation between PRNP expression and immunomodulators (immunostimulators and immunoinhibitors) was explored. We found that the top two positively associated immunostimulators were C10orf54 (Spearman *r* = 0.369) and TMEM173 (Spearman *r* = 0.267) (Supplementary Figure S1A), and the top two negatively associated immunoinhibitors were ADORA2A (Spearman *r* = −0.052) and VTCN1 (Spearman *r* = −0.041) (Supplementary Figure S1B). As shown in Supplementary Figure S2A, the correlation between PRNP expression and chemokines was explored. We found that PRNP was positively associated with several chemokines, such as CXCL17 (Spearman *r* = 0.255) and CXCL6 (Spearman *r* = 0.132). Additionally, we further investigated the correlation between PRNP expression and chemokine receptors and found that some receptors including CX3CR1 (Spearman *r* = 0.299) and CXCR1 (Spearman *r* = 0.241) were positively associated (Supplementary Figure S2B).

## 4. Discussion

Ferroptosis is involved in the tumorigenesis and progression of many cancers including ovarian cancer, figuring out the not-fully-interpreted molecular biomarkers, targets, and mechanisms underlying ferroptosis are of great value [33, 34]. In this study, three ferroptosis-related genes PRNP, ACSL1, and CP were picked out as the co-DEGs among different ovarian cancer datasets, with significantly decreased expression of PRNP and ACSL1 and increased expression of CP in ovarian cancer tissues when compared with normal ovarian tissues. Then we wonder if these three ferroptosis-related genes were strong enough to influence the prognosis of patients with OC. It only turned out that decreased expression of PRNP was associated with poor overall survival of OC patients. No prognostic role of the other two genes has been found. These accordance findings of PRNP in aspects of expression and survival in OC make us focused on PRNP and assume that PRNP might become a promising tumor-suppressive biomarker as well as a molecular target for further investigations. Up to now, we have not found any published paper exploring the role of PRNP in OC through the regulation of ferroptosis, while there were evidences of PRNP acting as an oncogene in other cancer types. For instance, overexpression of PRNP was reported to be associated with increased stemness of tumor cells in glioblastoma, pancreatic ductal adenocarcinoma, and osteosarcoma [35]. Moreover, PRNP in tumors promoted the transformation of mesenchymal subtype and associated with poor survival through Hippo signaling pathway in colorectal cancer [36]. NFIL3-PRNP axis has also been proved to increase the migration and invasion ability of lung cancer cells via JNK signaling pathway [37]. In addition, mechanistically, direct binding of PRNP to filamin A may exert the oncogenic role to promote the aggressiveness of tumor cells [38], and prognostic value to predict poor clinical outcome of digestive cancer has been issued [16, 19]. With the next step experiments in vitro showing the inhibitory role of PRNP in promoting the proliferation and invasion ability of OC, our study was the first to show the potential tumor-suppressive role of PRNP in OC, which is totally inconsistent with the oncogenic role of PRNP in other cancer types we mentioned above. This intriguing difference suggested the probably multiple and complicated regulatory mechanisms of PRNP for various types of cancer.

Next, we found that PRNP-associated functions were enriched in the Ras signaling pathway in OC by using KEGG analysis. Notably, it is well known that the activation of Ras-MEK-ERK signaling is indispensable for the induction of ferroptosis via elastin. In addition, taking into account that PRNP was identified as a ferroptosis-related gene in our study, it was rational to speculate that the tumor-inhibitory role of PRNP in OC patients might partially be through ferroptosis in a way dependent on Ras-MEK-ERK signaling pathway [39–41]. Future experimental exploration in this direction may be necessary.

The immune cells infiltration of tumor represents a particular pattern of the host immune reaction, thereby playing a pivotal role in tumor progression [42]. Increasing evidences have acknowledged the immune cell infiltration profile as an effective biomarker to predict the response of immunotherapies such as immune checkpoint inhibitors against PD-1/PD-L1 [43, 44]. In our study, we found that PRNP was positively correlated with infiltrated mast cells, T effector memory cells, and plasmacytoid dendritic cells. The previous studies demonstrated that mast cells expressed and released PRNP in response to inflammation [45], which interpreted our findings to some extent, and raised an intriguing question whether mast cell-induced inflammation (or other biological reactions) was involved in the tumor-suppressive role of PRNP for OC patients. Similarly, accumulation of PRNP located at the contact sites between T cells and dendritic cells has been reported [46]. Moreover, there was a significant association between PNRP and immunostimulators (C10orf54, TMEM173), immunoinhibitors (ADORA2A, VTCN1), chemokines (CXCL6, CXCL17), and chemokine receptors (CXCR1, CX3CR1). Summarizing the PNRP-associated immune infiltration profiles could help us better understand the heterogeneity of tumor microenvironment and guide the next step research.

However, there are still some shortcomings in this study. The function of PRNP was studied in this study, but the specific mechanism of PRNP producing these functions was not explored, and we will further carry out in subsequent studies. In addition, samples for the prognosis analysis were limited and need to be expanded in the future. Furthermore, most of the results and conclusions in this study were based on these online databases, and more experiments based on cells and tissues are needed to further verify them in future studies.

## 5. Conclusions

In general, this study demonstrated for the first time that ferroptosis-related gene PRNP exerted a tumor-suppressive role in OC and the aberrant expression and function of PRNP making it a potential novel biomarker for OC diagnosis, prognosis, and response to immunotherapies.

## Data Availability

The original contributions presented in the study are included in the article/Supplementary Materials, further inquiries can be directed to the corresponding authors.

## Supplementary Materials

Supplementary Materials Supplementary Figure S1. The relationship between PRNP expression and the immunomodulators in ovarian cancer. (A) The relationship between PRNP expression and the immunostimulators. (B) The relationship between PRNP expression and the immunoinhibitors. Supplementary Figure S2. The relationship between PRNP expression and the chemokine and its receptors in ovarian cancer. (A) The relationship between PRNP expression and the chemokines. (B) The relationship between PRNP expression and the chemokine receptors. Supplementary Table S1. The upregulated and downregulated genes between normal ovary and ovarian cancer tissues from GSE12470. Supplementary Table S2. The upregulated and downregulated genes between normal ovary and ovarian cancer tissues from GSE26712. Supplementary Table S3. The ferroptosis-related genes. Supplementary Table S4. Logistic regression analysis of PRNP expression correlated with clinicopathological factors in ovarian cancer.
