# Supplementary material for: Downregulated PRNP Facilitates Cell Proliferation and Invasion and Has Effect on the Immune Regulation in Ovarian Cancer
Source: J Immunol Res. 2022 Sep 29;2022:3205040. doi: 10.1155/2022/3205040 (PMC9537007; doi:10.1155/2022/3205040)
Supplement: Supplementary Materials — Supplementary Figure S1. The relationship between PRNP expression and the immunomodulators in ovarian cancer. (A) The relationship between PRNP expression and the immunostimulators. (B) The relationship between PRNP expression and the immunoinhibitors. Supplementary Figure S2. The relationship between PRNP expression and the chemokine and its receptors in ovarian cancer. (A) The relationship between PRNP expression and the chemokines. (B) The relationship between PRNP expression and the chemokine receptors. Supplementary Table S1. The upregulated and downregulated genes between normal ovary and ovarian cancer tissues from GSE12470. Supplementary Table S2. The upregulated and downregulated genes between normal ovary and ovarian cancer tissues from GSE26712. Supplementary Table S3. The ferroptosis-related genes. Supplementary Table S4. Logistic regression analysis of PRNP expression correlated with clinicopathological factors in ovarian cancer. [file 3205040.f1.zip › Supplementary Table S1..docx]

**Supplementary Table S1.** The up-regulated and down-regulated genes between normal ovary and ovarian cancer tissues from GSE12470.

| **up-regulated genes in GSE12470** | | | | | |
| --- | --- | --- | --- | --- | --- |
| TMEM139 | COL7A1 | GBP5 | MED14 | ZNF273 | AK2 |
| C16orf59 | IMPA2 | LAMA5 | PPOX | PTCD1 | MRE11A |
| RASSF7 | ZNF404 | SLC39A11 | C8orf33 | TMEM79 | TBC1D7 |
| TROAP | CDH24 | PABPC3 | ZNF92 | HRASLS | NHP2 |
| C19orf33 | DNALI1 | CIITA | PTCD2 | EZR | STK36 |
| OIP5 | PLLP | WHSC1 | LSG1 | GPT | RPP38 |
| KIFC1 | VCY | DGAT1 | DNAAF2 | CITED4 | KRT17 |
| ASPM | CIART | ATP5B | ILF2 | RANBP1 | FASTK |
| TMEM125 | DOK5 | ETV7 | CLPB | SEPHS1 | PROSER1 |
| CDCA5 | SAA2 | MARK2 | DLGAP1-AS2 | HIST1H2AG | GLB1 |
| TSTD1 | RCC1L | SLF1 | FAM173A | ZNF443 | APOBEC3B |
| ESRP2 | ROPN1L | UCK2 | LPAR2 | EIF6 | BRMS1 |
| MREG | TRPV6 | DENR | DBF4B | CLEC2D | TTLL4 |
| CENPM | IGLL1 | HLA-DOB | ZNF581 | ZDHHC12 | YARS2 |
| KRTCAP3 | HP | TATDN1 | GRHL1 | LEAP2 | FBXO46 |
| KRT18 | TMEM63A | TFF2 | SLC37A1 | HINT2 | ELL3 |
| CDC45 | NELL2 | TTC21A | GPN2 | MBD4 | STMN1 |
| BLM | PRR15 | LRRK1 | ZXDC | DDT | TMEM61 |
| GINS2 | TSPAN12 | KLK1 | PON2 | PUS7 | LY6E |
| DSP | ISG20 | TMEM8A | ASAP2 | MORN3 | MCM6 |
| NCAPH | C2 | VCX2 | MRI1 | APEX1 | CIB1 |
| GPT2 | SIGLEC7 | GIGYF1 | NUP37 | PLPP2 | CLDN23 |
| CRB3 | XAF1 | NCOA3 | STRA6 | ZNF195 | WDR4 |
| FANCA | STAT2 | PIM2 | RPL15 | PTPN6 | IQCC |
| PKMYT1 | PLPPR3 | AIFM2 | ING4 | RASGRP1 | CDC7 |
| NUF2 | DDIT4 | ZMYND19 | HSCB | XPO5 | MKRN1 |
| RHPN2 | MYLK2 | APOBEC3G | RTN4R | LRTOMT | KDM4A |
| LINC01138 | SERPINA1 | CCDC6 | CHST4 | TRAPPC9 | TMUB1 |
| MYBL2 | GPAA1 | ARL6IP6 | STX16 | HILPDA | EFNA1 |
| ORC6 | KLHL6 | FOLH1 | REXO4 | RHNO1 | USP45 |
| NCAPG | NPAS3 | ZBTB7B | ZNF782 | SMARCA4 | THEM6 |
| MGME1 | PVALB | POT1 | HDGFL1 | PHOSPHO2 | MRM1 |
| DDR1 | ICAM5 | PIK3R2 | TRMT13 | PYCR1 | TMEM14B |
| RECQL4 | APLN | PDSS1 | C11orf54 | NUDT1 | VRK1 |
| PTTG2 | APBA2 | ZFP69B | SLC39A10 | ALG1 | POMGNT1 |
| C10orf95 | RBP1 | C1orf159 | LRRC14 | CENPBD1 | MTG1 |
| DENND2D | LHX1 | DBP | REC8 | TPD52L1 | FUK |
| PAFAH1B3 | TGFA | ATG4D | GTPBP10 | H2AFV | PHKA2 |
| UBE2T | C20orf85 | SRSF6 | LOC401127 | NRBP2 | ICE2 |
| EPS8L1 | NME5 | TGIF1 | XAGE5 | NARF | FOXRED1 |
| CDT1 | EGLN3 | GATAD1 | TRIB3 | SUMF1 | TRIM24 |
| POC1A | NXF3 | BCL2L1 | NUDCD1 | RPLP0 | SSSCA1 |
| ELMO3 | PIK3IP1 | FUOM | RORC | DDX39A | SPOCK2 |
| ECE2 | POU2AF1 | CYP3A5 | TAPBP | UGT2B10 | HAUS6 |
| ANLN | ENKUR | CPAMD8 | NME6 | LYPD6 | ORC5 |
| SH2D3A | TNNI2 | MRPS23 | F8A1 | FBXO21 | HTATIP2 |
| MCM9 | HLA-DQA1 | HINT3 | TSPAN1 | MRPL23 | CSTB |
| PARPBP | RNF182 | KRT5 | ZNF507 | ALDH7A1 | FAM200A |
| LRRC61 | FCGR1A | IARS | PLXNB1 | ALDOA | ZC2HC1C |
| CHMP4C | COL10A1 | IFT74 | TMEM183A | NEK11 | E2F3 |
| EME1 | UQCRH | TTLL12 | ZNF202 | ZKSCAN4 | NCOA2 |
| ZNF587 | IRF5 | STC2 | RPAP3 | ADAM28 | PDZD11 |
| HOOK1 | HOXB6 | NRL | DERL1 | RPL39 | OGFOD2 |
| KRT19 | ZNF750 | REEP4 | DCBLD2 | CEP70 | COQ9 |
| B3GNT3 | LIMS3 | PPFIBP1 | KPNA2 | TNFSF13 | SORL1 |
| C1RL | ZNF214 | PILRA | ACP1 | DNAAF5 | IFI30 |
| CTSV | C20orf196 | G3BP1 | TADA1 | EPSTI1 | UQCC1 |
| TM7SF2 | CLDN1 | AFG3L2 | NLK | ATP1B1 | SARS2 |
| RAD51 | C5orf46 | DIAPH1 | MSTO1 | H3F3A | RAB26 |
| RAD54B | LRG1 | SUV39H2 | PSMA5 | SCD | SLX1A |
| VAMP8 | WNT6 | ALS2CL | OSGEPL1 | PSMA6 | SH3RF2 |
| DDIAS | TMEFF1 | MOB3B | YIPF3 | ANAPC1 | C11orf49 |
| CEP72 | GDF15 | KCNN4 | EXOSC2 | PXMP4 | ACTR3B |
| CDH1 | NETO2 | NRAS | ZNF589 | ZNF823 | CAPS |
| SCNN1A | CPNE7 | ZNF296 | PAQR6 | STAG3 | NCBP2-AS2 |
| KIF15 | GAD1 | MLF1 | ATL3 | HEATR1 | TBL1XR1 |
| RRM2 | SIAE | GGH | NINL | LNX2 | GSTK1 |
| CENPA | IRX3 | LMBR1 | TEX10 | RHBDD3 | TMEM186 |
| EVPL | HGD | PRKAB1 | JMJD4 | INHA | FDPS |
| BORA | UTS2 | PTGER4P2-CDK2AP2P2 | TTPAL | ARFGEF2 | LOC728743 |
| RHPN1 | KCNK13 | ATF7IP2 | AMY2A | TUFM | AVL9 |
| PPP1R9A | PKP3 | TTC9 | CDC23 | ATP2C1 | OPN3 |
| TRAIP | SPINT2 | ANXA3 | SNRNP25 | CPNE3 | TST |
| GRB7 | EPCAM | PEG10 | C11orf71 | R3HDM1 | ERMP1 |
| ARHGAP8 | CLDN4 | GPSM2 | FBXL2 | PODXL2 | C12orf65 |
| PKP2 | CLDN7 | IFI44L | RPRD1A | DCAF17 | IQCA1 |
| CHTF18 | PRSS8 | FAF2 | KCNK5 | MZT2B | SPSB2 |
| PLK4 | TK1 | PGD | KRI1 | MRPL14 | MVK |
| STRBP | UBE2C | ZNF397 | ERCC6 | MAST1 | GPR180 |
| EZH2 | CCNB2 | MFSD13A | GRAMD1A | SKAP1 | TMPO |
| HMGB3P1 | BIRC5 | TUBG1 | MBOAT7 | TMC6 | PATZ1 |
| MCRIP2 | CENPF | PIGF | ARMC7 | CDK4 | EXOSC3 |
| FAAH2 | BUB1B | ZNF124 | TGDS | PPIH | HIST2H4B |
| MKI67 | TRIP13 | CD2AP | TBC1D3F | PPA1 | SYK |
| KIF22 | DHCR24 | TIGD2 | OGT | 44626 | MLXIPL |
| KIAA0907 | PAX8 | FDXR | SRD5A3 | BABAM1 | FADD |
| CDS1 | KIF2C | TAS2R10 | ZNF514 | PUS1 | MRPS16 |
| ARHGEF5 | CDCA8 | ANKMY1 | EML4 | IQCB1 | ESRRA |
| HIST1H2AK | CDK1 | NQO1 | PPM1G | NAPG | DHTKD1 |
| RAB3IP | CLDN3 | ZNF763 | IFITM1 | FAM187A | QTRT2 |
| DBNDD1 | KRT8 | FGFR2 | RCN2 | STOML2 | SLC35F3 |
| KDF1 | AURKA | PPIL2 | SUV39H1 | FANCL | ZNF607 |
| ZDHHC23 | CDC20 | MLST8 | MOCS3 | MASTL | GEMIN7 |
| IGF2BP2 | MCM2 | MYBPC2 | IL23A | MRPL19 | EEF1E1 |
| C17orf53 | MCM4 | ZNF19 | ADAM10 | ACAD9 | ZNF550 |
| MSI2 | FAM64A | CDK3 | SRD5A1 | EPOR | OARD1 |
| CENPE | TPD52 | SH3BP2 | NAA50 | MDH2 | PARP12 |
| MRS2 | KIAA0101 | ABCC6 | HIST1H3F | GOSR1 | MCCC2 |
| NMU | PTTG1 | FLVCR2 | MKS1 | FBXO6 | DKC1 |
| KLK10 | KLK8 | WDSUB1 | COX18 | HIGD2A | CORT |
| LAMB3 | CCNB1 | ATP6V1A | ARL4C | ATP23 | ZMYM3 |
| TMEM177 | SLPI | CPOX | MICB | FAM24B | NFX1 |
| INCENP | KIF20A | CD22 | PRICKLE3 | TERT | TMED10 |
| C10orf35 | SLC2A1 | HLA-DRA | FIGNL1 | SLC5A6 | RFT1 |
| TLCD1 | ELF3 | VILL | ATXN2L | TRUB1 | PSMD2 |
| NIPSNAP1 | STXBP2 | IRAK1 | HCN3 | POLG2 | LMNB2 |
| RTKN | NUSAP1 | STXBP6 | MYBPH | PAK4 | DTX3L |
| PDZK1IP1 | SLC39A4 | FGF18 | ZNF552 | HSH2D | PIGC |
| ZNF572 | ST14 | ARHGAP4 | ACBD5 | APOOL | USP28 |
| HMGB3 | PSAT1 | SDHAF3 | TTC26 | MTHFS | PRIM1 |
| FBXL16 | SPP1 | MYCN | PUDP | ZDHHC9 | GINS3 |
| BDH1 | KLK6 | TRIM45 | DBR1 | SMYD2 | ALKBH4 |
| OR7E24 | ESPL1 | UBXN11 | PEMT | ILVBL | CPSF3 |
| DHCR7 | FOLR1 | METTL18 | SLC2A5 | ADNP | CA14 |
| KLK11 | ASF1B | VPS37D | BNIP3 | MUTYH | ZDHHC21 |
| TNNI3 | PRC1 | ANKRD6 | CXCL16 | NARS2 | C5orf42 |
| SLC25A10 | ABHD11 | VAV3 | WRNIP1 | SCAMP5 | BRD8 |
| NCAPD2 | CENPU | DCP2 | TASP1 | ITGA3 | ZNF579 |
| CREB3L4 | CKS2 | OR7E19P | ADAMTS13 | C11orf1 | TRMT2B |
| TYMS | APOC1 | CLEC18B | HLA-DMB | ZSCAN9 | MFN1 |
| HIST1H2AE | MAD2L1 | NEDD4L | JTB | DNTTIP1 | FASTKD3 |
| NFE2L3 | SDC1 | RBL1 | ZFPL1 | HIST1H3E | MPLKIP |
| SHTN1 | GRHL2 | KIF21A | CARD9 | IL1RAP | RPUSD2 |
| HIST1H2AB | HIST1H2BD | PMAIP1 | ANKRD49 | EIF2AK2 | BEX2 |
| L2HGDH | RACGAP1 | DEPTOR | HMGN3 | AZIN1 | ZNF440 |
| ZBED6CL | DTL | ENC1 | NDUFAF6 | SLC52A3 | GTF2H4 |
| ATAD2 | LSR | BCAS4 | SOX4 | SLC16A3 | ILF3 |
| ACY1 | AHCY | LINC00839 | NXT2 | DYX1C1 | APOL4 |
| ITGB8 | PTPRF | VBP1 | ZKSCAN7 | VMA21 | TUBGCP4 |
| DNMT3B | BIK | C15orf39 | SUGP1 | LAMTOR5 | OSBP2 |
| ETV4 | CBS | CDR2L | THUMPD3 | CENPL | GGCT |
| RGL3 | CCNE1 | TRAF7 | GMPR | UPK3B | C8orf76 |
| CCDC150 | ECT2 | MX1 | MEN1 | STAG3L3 | C19orf52 |
| TMEM69 | TMPRSS4 | SERPINA5 | LIMK2 | CRLS1 | C16orf70 |
| MOGS | PAM16 | TLE6 | HLTF | ZNF442 | CREBZF |
| C19orf48 | TIMELESS | SLC35F5 | RAB36 | C14orf79 | UQCRC1 |
| PSD4 | TACC3 | CXCL9 | C1orf109 | DNASE2 | MTERF1 |
| MAP7 | SORD | NME9 | OSBPL2 | RDH13 | UPF3B |
| MUC20 | LSM4 | ADGRG5 | LYPLA2 | SLC12A8 | RPUSD3 |
| CELF4 | PSRC1 | FTCD | SLC25A5 | STX1A | WDR54 |
| RMI2 | UCP2 | MTPN | ST7 | DECR2 | GMPS |
| FAM136A | PDIA4 | PITPNM1 | SDF2L1 | GGA3 | PRPF40A |
| KNSTRN | MUC1 | H1F0 | DCTPP1 | OR2A9P | ITCH |
| ARHGEF16 | SFN | SMCO2 | RHBDD2 | CTPS2 | HSD17B7 |
| C15orf48 | MDK | PNMA5 | TNFSF10 | GPR160 | KLHL35 |
| FOXJ1 | HDGF | ANKRD18A | TAPBPL | RNASET2 | TTC30A |
| TOM1L1 | SCRIB | NDC80 | TFCP2 | GNG5 | RRAGD |
| UHRF1 | MCM7 | PSORS1C1 | ARRDC1 | TUFT1 | NDUFS1 |
| PCK2 | SHMT2 | SLC35E1 | FAM86B1 | PALB2 | AGTRAP |
| EDEM2 | NCBP2 | ZBTB44 | ZNF185 | METTL6 | RAD21 |
| TMC4 | MIF | ITPKA | AMY2B | RMDN1 | TRIM52 |
| LRRC8D | COX5B | ZNF232 | CBR4 | ABHD3 | ZNF518A |
| PCBD2 | RFXANK | TSPOAP1 | NDUFAB1 | CHTOP | GALNT2 |
| LARGE2 | GSTP1 | CHDH | SLC29A3 | NUDT6 | RPS2P32 |
| E2F5 | JUP | SPAG8 | N4BP3 | MRPS18A | P2RY6 |
| SLC4A11 | ABCF3 | DDX60 | AMY1C | ZNF74 | XRCC1 |
| SPAG5 | LAMP3 | ZNF285 | WDR83 | RAD52 | PPFIA1 |
| SGO2 | RNASEH2A | GMFB | ASB7 | ASTE1 | RNF5 |
| NDC1 | HIST1H1C | PRRC1 | IQCG | PRIM2 | SFXN4 |
| C9orf116 | CDKN3 | STXBP4 | CEP89 | C18orf25 | WARS2 |
| RNFT2 | PRKCI | SLC37A2 | C8orf44 | MT3 | PRMT6 |
| TMEM41A | HIST1H2BH | SNHG7 | CCDC191 | SYNGR3 | ABHD16A |
| ACOT11 | MRPL12 | HES6 | NIT2 | USP30 | ALAD |
| EARS2 | LAPTM4B | GBP3 | NUS1 | CASP6 | LAGE3 |
| EFNA4 | EXOSC4 | SAMD9L | PEX6 | YEATS4 | MRPS18B |
| TDP1 | GPI | ALYREF | LYG1 | ZNF687 | PRRT3 |
| ZNF485 | CDKN2A | POC1B | HIST1H4K | ETNK1 | MCOLN2 |
| SLC9A7 | PRSS2 | LDAH | PMS2 | C1orf131 | RIBC1 |
| HIST1H3B | MRPS12 | NECTIN3 | RAB6B | BROX | PPAT |
| PACSIN1 | ASS1 | COPS7B | RARRES3 | GON7 | MMP1 |
| POLR2H | PRSS21 | SEC14L4 | TARDBP | RRP9 | NKRF |
| DARS2 | CAPG | GIPC1 | EDEM1 | GPRC5B | UBD |
| PPP1R16A | ASRGL1 | IFIH1 | PUSL1 | CCDC57 | PPM1J |
| ZNF681 | PKM | C1orf226 | FGFRL1 | HSD17B1 | FLJ23867 |
| ZNF430 | TSTA3 | SPRY3 | WDR45B | PGBD3 | ITGB7 |
| CDC6 | FARSA | ARTN | C22orf29 | KATNB1 | APEX2 |
| SYT17 | SCGB2A1 | IFI44 | BET1 | STRADB | TCF7 |
| NSUN5P1 | GAPDH | FAM222A | TTC13 | MRPL11 | MANEAL |
| PBX4 | TPI1 | WBP1L | CROCCP2 | RPS21 | SNRPD3 |
| SH2D4A | SMARCC1 | KRT14 | PCMTD2 | PREPL | ZNF7 |
| PPP1R14C | HN1 | GDF11 | MBD6 | MTMR1 | C19orf57 |
| NOXO1 | CHI3L1 | FBP1 | GMDS | WDR3 | RAB42 |
| EPS8L2 | MRPL13 | ASPHD2 | TMEM106C | ZC3H8 | RPP25L |
| MGAT4B | IDH2 | TNF | CHCHD1 | MAGT1 | TMEM267 |
| MFSD3 | CALR | PLA2G7 | EFHC1 | IKBKB | VPS72 |
| OPLAH | ISYNA1 | FAM26F | PARP9 | PNPLA4 | TMEM38A |
| ZNF85 | CCT5 | ARID2 | ASUN | RPUSD1 | DHRS2 |
| ZNF107 | SORT1 | COMMD2 | SEC23B | GTF2H3 | LRRC1 |
| GPD2 | PPIF | LRRC46 | LGR6 | MRM2 | DIRC2 |
| POLA2 | RAE1 | CTNND1 | BPHL | BHLHE41 | HDAC1 |
| SEZ6L2 | PUF60 | GRTP1 | BID | CARMIL1 | NUP107 |
| DSG2 | PYCRL | FGGY | REPS2 | CCDC15 | HIST1H3A |
| CHKA | DEFB1 | ALOX5 | DTYMK | CCT3 | PYGO2 |
| KLHL24 | CD24 | ZNF234 | SENP5 | AAAS | HACD3 |
| LBHD1 | MECOM | BEND7 | PAFAH1B2 | VPS25 | NCK1 |
| RAB11FIP4 | HIST1H2BE | PLXNB2 | NRIR///CMPK2 | PSME1 | KCNE3 |
| ZSCAN16 | SCGB1D2 | ARF3 | HEXIM2 | CAD | ZNF419 |
| DAPL1 | LRFN4 | KIAA0319L | AASDHPPT | HIPK2 | ARV1 |
| HN1L | EIF4EBP1 | HID1 | WHSC1L1 | IPO4 | RBM19 |
| POLD1 | CYBA | TSNAXIP1 | NPEPPS | VKORC1L1 | SPA17 |
| DTNB | SLC50A1 | GYG2 | ZNF20 | PSMC4 | ZMYND10 |
| HOOK2 | MRPL2 | DDX58 | TCTEX1D2 | CCDC125 | HMGB2 |
| HPDL | MARCKSL1 | ERFE | GOLGA8B | PRDX2 | TARBP1 |
| IL4I1 | PPP1CA | LINC01315 | LYPLA1 | HIST1H4J | SLC35F2 |
| APEH | RPN2 | MOCOS | WDR48 | ZNF700 | PCGF1 |
| IGSF9 | TRAF4 | XXYLT1 | CTSB | HENMT1 | WDR5 |
| CDCA7 | COX7B | GLMP | MRPL28 | EBNA1BP2 | AP5S1 |
| ZNF493 | MPZL2 | POLA1 | SUPV3L1 | NMRAL1 | ACTL10 |
| RBM47 | PPP1R14B | SMPD3 | NUDT14 | HOMEZ | ZSCAN25 |
| C11orf52 | ST6GALNAC2 | SIGLEC15 | KIF2A | BAIAP2 | SLC25A38 |
| CKAP2 | SOX9 | SCG5 | RPS19 | PRMT3 | SS18L1 |
| MIS18A | TECR | MYBL1 | TMEM206 | LETMD1 | POLR3E |
| AQP5 | BAK1 | CYP3A7 | TMEM187 | MED13L | ANKRD65 |
| CHAF1A | RBM38 | ANXA9 | PPAN | ASL | ANKHD1-EIF4EBP3 |
| MTIF2 | COX8A | NRTN | ELMOD2 | WDR77 | TOP1MT |
| CLCN2 | ERBB2 | HOXB8 | DRC1 | BMF | BCAP31 |
| EBP | ADGRG1 | IFIT1 | LARP1B | ST3GAL6 | PUS10 |
| ITPR3 | SLC52A2 | CBL | WDR66 | MRPL47 | TMEM9 |
| ZNF165 | FIBP | OAS2 | CA8 | PHB2 | FBXO2 |
| DDX11 | MCM3 | LPAR5 | TOMM40 | FUNDC1 | ORC3 |
| TMEM223 | NDUFB11 | CFAP44 | BATF2 | ZW10 | KDM1A |
| RASAL1 | H2AFX | SLC13A5 | KAT14 | GALM | MAGIX |
| HIST1H2AL | PDCD5 | LDHD | METTL2B | FLVCR1 | LRRC45 |
| UBFD1 | FBXL6 | PIGW | NCBP1 | PTPRS | HIST1H2BB |
| C11orf80 | FKBP4 | SAMD9 | ZNF526 | CYP4B1 | DNAJC30 |
| ZNF138 | POLR2I | GPC2 | TMEM260 | METTL21A | FNBP1L |
| LAMC2 | NDUFA7 | INA | FOLR3 | ECHS1 | IQCK |
| GALE | KRT23 | AK4 | NIPAL3 | FMR1 | CDCA3 |
| SLC9A3R1 | NR2F6 | TNPO3 | CPSF1 | EIF2AK1 | SMC5 |
| LIG1 | POR | SDS | PTER | NLN | COL23A1 |
| LLGL2 | TUBB | PGAP2 | ZNF564 | ZNF57 | DNMT1 |
| FAAH | UQCR10 | HIST1H2BF | ZSWIM3 | AK8 | BOLA1 |
| RAC3 | TACSTD2 | FBXW9 | COA7 | DNASE1L2 | EEF2KMT |
| ZNF429 | ATP6V0B | C20orf96 | ETAA1 | ECHDC3 | PEX11A |
| NSUN5 | SNRPB | PTPN3 | PSMB10 | NTPCR | CHST6 |
| SDSL | TKT | PROSER2 | TMEM87B | SEMA4G | PAK1 |
| SMPDL3B | S100A2 | ATP5J2 | OR7E5P | MIS18BP1 | FHIT |
| HGH1 | RPL39L | FAM122B | PTBP3 | MRPS2 | GPX1 |
| MAPK13 | PPP4C | TBRG4 | TTC32 | GCSH | DNAJC19 |
| TARBP2 | ENO1 | MESP1 | SLAMF8 | XPOT | MEA1 |
| EXO1 | MRPL4 | GATC | SLC33A1 | ZBED8 | HDDC3 |
| FEN1 | MYO10 | RUVBL1 | WDR78 | PC | GOLPH3L |
| GCHFR | BZW2 | GTF3C3 | NEUROG3 | SPTBN2 | DBF4 |
| GOLT1A | RPN1 | MCM3AP-AS1 | PSTK | UBE2Q1 | CEP41 |
| THOC3 | PRKCD | ABI2 | CEP76 | DCAF4 | IPP |
| ZNF675 | COX6B1 | ZNF43 | TBC1D2 | HMGN1 | MYO6 |
| ZNHIT2 | PPDPF | MYCBP | CAPSL | URI1 | OLA1 |
| WBSCR27 | ATP5G3 | PRDX5 | PHGDH | ATP5S | NUDT21 |
| PRRG4 | AURKAIP1 | GRAMD1C | IL20RB | CYP2D7 | NAXE |
| ZNF714 | TRIM27 | OBSCN | ATP7B | CLIC3 | TMEM205 |
| TMCO6 | COL9A2 | CENPO | ZNF608 | GTF2E1 | BCKDHA |
| MSH5 | COPG1 | CTSA | SPDEF | GPR107 | METTL3 |
| CMTM7 | C19orf53 | COA4 | KIF3B | MRPS21 | AREL1 |
| HIST1H1E | NTHL1 | FZD5 | ZNF300 | NSDHL | PTGR2 |
| GMNN | MT1F | NIF3L1 | SLC2A11 | PDHA1 | ZNF512B |
| ZNF486 | CKS1B | MRPS33 | HYAL3 | PRDM15 | ARL17A |
| EXOSC5 | CARHSP1 | AMPD3 | TIMM10 | HPS3 | ALG10 |
| CARD14 | ISG15 | OR8B8 | DLG1 | LIMK1 | PRR13 |
| TTC30B | MAL | PARS2 | MCUR1 | RNF34 | IFT22 |
| KLF5 | IRF9 | SHMT1 | DDOST | IFT140 | CLN6 |
| FAAP24 | CHODL | HKDC1 | ZNF44 | ARFGEF1 | GALNT12 |
| NOXA1 | AGRN | REPIN1 | SULT1A2 | CBX3 | VANGL1 |
| HIST2H2AA3 | DNPH1 | LINC01106 | ADPRHL2 | NUP155 | FANK1 |
| RITA1 | IFI27 | AP1G2 | RAD50 | FAM76B | GFER |
| HIST3H2BB | SMG7 | NDUFS8 | GSDMB | MTHFSD | TOR2A |
| HIST1H2BN | S100A11 | EGFL6 | C5orf22 | RPL37 | HACD2 |
| HSD17B8 | LAMTOR2 | HIST3H2A | KIF1B | SPEF2 | USP21 |
| ZMYND12 | LSM8 | DPP3 | CMTM6 | ENOSF1 | ALG6 |
| CLUHP3 | NME1 | FAM110A | RNASEH1 | CLN3 | TRIM11 |
| MCM8 | HYOU1 | RCC1 | VPS33B | POLE | ZNF839 |
| ZNF506 | DCXR | TTC12 | FAM103A1 | CELF1 | MIPEP |
| LINC00467 | NDUFA13 | ACP6 | CELSR2 | MOV10 | POP7 |
| ANKZF1 | HIST1H2BK | ZNF250 | DOC2A | TFR2 | PIGM |
| KLHDC9 | NDUFA3 | PCNA | GLO1 | KIFC2 | COA5 |
| USP18 | MSH6 | FAM96A | CISD2 | TCF3 | NDUFB9 |
| PHKG2 | WBSCR22 | QPRT | SSU72 | TMED4 | GTPBP3 |
| MYEF2 | PGLS | PRSS22 | HIST2H2AC | ZNF16 | MAP6D1 |
| CHAF1B | PCYT2 | SH3YL1 | GLE1 | RNF170 | GMIP |
| DNMT3A | IGFBP2 | TIGD5 | B4GALNT4 | TFB2M | RANBP17 |
| TBC1D31 | RUVBL2 | ZNF217 | PNMA3 | CEP85 | RFC2 |
| SGPL1 | MID1IP1 | ZNF692 | ZZZ3 | ZNF14 | PRICKLE4 |
| FAM60A | UBE2S | ERGIC3 | RNF2 | MRPS30 | TMBIM6 |
| ASPHD1 | RBBP4 | PDRG1 | FUT5 | MTHFD1 | SUGP2 |
| LINC01089 | UBAP2L | PSENEN | MAGED1 | NUPL2 | YEATS2 |
| DMKN | SYNGR2 | HDHD3 | TMEM251 | ADIPOR1 | TFPT |
| C6orf136 | SMC4 | NELFCD | PDIA5 | UBXN10 | TRIM14 |
| MSH2 | KLK5 | RSRP1 | HIST1H4I | MFSD4B | COMMD9 |
| BMS1P5 | PNN | APOBEC3D | BHLHA15 | RCOR3 | MRPL21 |
| TARS2 | S100A13 | FASTKD1 | SUSD3 | IGFLR1 | FARP1 |
| SMPD2 | LAD1 | TMTC4 | ASNA1 | FZD6 | ATF7IP |
| SNRPC | BMP7 | CORO2A | GOT2 | PLCXD1 | NECAB3 |
| OXNAD1 | BST2 | SMUG1 | PRR12 | PSPH | NPRL3 |
| RMI1 | PSMB2 | WDHD1 | API5 | PNMAL1 | PLEKHB2 |
| LRP8 | LGALS3BP | TTF2 | TFAM | PDCD10 | ERO1A |
| TMEM164 | NUP62 | TGM1 | DLAT | MPHOSPH9 | GCNT1 |
| PARP4 | GCAT | CTAGE15 | EHMT2 | LY75 | F12 |
| KIF23 | AKAP8L | SFXN1 | PANK3 | CYHR1 | CCDC51 |
| RTP4 | TUBA4A | MTA3 | DNAL1 | WDR53 | USP54 |
| OXLD1 | MMP7 | ZDHHC16 | DDX11L5 | C2orf49 | FUBP1 |
| WNT10A | IFI6 | KRT15 | TSPAN3 | CCDC167 | HARBI1 |
| PRKX | CAPN1 | CPT2 | HYLS1 | ACOT4 | FAM91A1 |
| NAA40 | CD74 | OCIAD2 | ZNF503 | GID8 | CIAO1 |
| ZNF117 | H1FX | HIST1H2BC | VPS33A | BPNT1 | FAM213B |
| FAM199X | STIP1 | UNC93B1 | ATP5C1 | EVA1A | PDIA6 |
| SAC3D1 | DCAF15 | ACOT8 | C17orf75 | NUDT8 | EDARADD |
| SYTL1 | GCDH | PIGO | RSPH14 | CXXC5 | ZNF606 |
| C1orf112 | CXCR4 | STAP2 | ATP13A1 | CMTM8 | ZNF627 |
| AIFM1 | VEGFA | CEP250 | UPF1 | HPSE | ENTPD6 |
| ZNF678 | MTHFD2 | HIST1H2BI | UHMK1 | U2SURP | NDUFB5 |
| ZNF208 | GSDMD | APOO | RPS6KA1 | NMI | DNAJA3 |
| FZD3 | PAK2 | DCLRE1C | HSD11B2 | ZNF786 | P2RY2 |
| KNTC1 | MAZ | SRPK1 | VPS52 | NUDT16L1 | TRAF2 |
| PRKCZ | CXCL13 | ZNF239 | ADGRA3 | YPEL1 | EFCAB11 |
| SLC25A39 | NT5DC2 | HIST1H2BO | CD46 | RPE | DHRS13 |
| FANCF | THEMIS2 | CSNK2A1 | RNPEP | POP5 | MRGBP |
| SLC37A4 | AGPAT2 | BRAF | VMP1 | GNL3L | HMBS |
| STOX1 | KLHDC3 | MCCC1 | SLC25A24 | DIP2B | MRPL35 |
| TMEM132A | GRINA | PARP1 | BOP1 | PEX11B | SLAIN1 |
| PAQR4 | PRKCSH | LMNB1 | MAP3K13 | LMLN | C1orf186 |
| MEST | RANGAP1 | NUDT15 | XPO4 | DPH2 | METTL13 |
| CELSR1 | OASL | BSCL2 | NBN | COPB2 | ENO3 |
| LOC100506282 | CTSD | DCLRE1A | FAM182B | BCL2L12 | PNPT1 |
| MCM5 | TUBB3 | OSBPL3 | ZNF563 | SPAG4 | INPPL1 |
| REST | RGS1 | MST1R | ST6GALNAC1 | CUL7 | IDO1 |
| NCAPG2 | APOE | CFAP70 | SEPSECS | H2AFY | OTUD6B |
| PDK1 | CRABP1 | GTF3C2 | DIEXF | SLC25A13 | HIST2H4A |
| ARMC10 | GANAB | IQCD | ABHD12 | MRPL48 | FRAT2 |
| CCL28 | OXTR | COA6 | LRIF1 | BICD1 | TLE1 |
| ALG3 | STEAP3 | PSMG3 | MBTPS2 | NR2C2AP | SNRPA |
| HPN | GSS | WDR34 | TNFRSF21 | FLAD1 | HSPE1 |
| GLS2 | SLC11A2 | CCDC142 | DIDO1 | RIBC2 | POLR1B |
| PPP1R35 | TFRC | COASY | MTM1 | ZNF626 | C7orf49 |
| SPDL1 | CP | TRIM17 | OSCP1 | MAGEF1 | MAPKAPK3 |
| PLA2G12A | ZDHHC13 | RBKS | UBQLN4 | MRPS35 | TMEM102 |
| ACTL6A | WDR5B | LMAN1 | ADSSL1 | HIST1H4F | MOSPD3 |
| POU5F1 | FKBPL | CXCL10 | ZNF600 | PPARGC1B | STAT1 |
| C5orf30 | ZNF707 | KIAA1551 | CKLF | STYXL1 | MRPL57 |
| HIST1H2AD | CD47 | WDR12 | MIEF2 | SLC25A29 | CDCA4 |
| USP6NL | CAPN13 | TUT1 | CENPQ | SIPA1L1 | MRPL3 |
| SLC25A22 | SLC16A5 | ZMYND8 | POLR3A | KMT5C | ECI1 |
| OAS3 | GRHL3 | CSNK2B | P4HA1 | ANKRD16 | RAP1GAP |
| RSPH3 | ATIC | RCCD1 | DUS1L | TOPBP1 | IFT122 |
| TAF4 | DNAH7 | HINFP | SCD5 | HIST1H2AH | IGF2BP3 |
| RPA3 | APITD1 | CSTF1 | REEP6 | LARP4B | ATR |
| SPICE1 | WDR62 | PCBD1 | ENOPH1 | C12orf60 | PPP1R26 |
| DERL3 | ZNF3 | GET4 | TRPV1 | PTPMT1 | PAIP1 |
| TMEM209 | CBFA2T2 | CFB | FUZ | ZWILCH | ORMDL2 |
| ZNF320 | NPHP3 | ERP27 | SC5D | FAM181A | TRPT1 |
| PFDN6 | TIMM17B | TXK | PTPN18 | DNAJC9 | MNS1 |
| TTC9C | HIST1H3H | PROM2 | EMC10 | HSPB11 | KCTD1 |
| IDH1 | GJB1 | PRR7 | ELOVL6 | SLC22A18 | VARS2 |
| CYB561 | FCGBP | COMTD1 | SPATA2 | TFDP2 | ZFP64 |
| NUP43 | WDR91 | TMEM92 | FDFT1 | C1GALT1 | C14orf93 |
| ZNF816 | CDK5 | MRPL17 | PHACTR4 | CXorf38 | FGFR1OP |
| RFC4 | UCKL1 | LACTB2 | YARS | SFT2D3 | APRT |
| SCGB1D1 | TRUB2 | NUDT22 | CDCA7L | MCAT | SLC25A16 |
| DPY30 | RHBDF2 | TRAP1 | SHQ1 | SLC16A6 | HIST1H4H |
| NDUFV1 | ZNF48 | TMEM33 | ATP6V1C1 | QARS | TPGS2 |
| VAC14 | OXSM | CENPH | TMEM231 | DPCD | AIMP2 |
| SSC4D | LMO7 | SWSAP1 | GALNT7 | HAGHL | GART |
| GPRC5C | LONP2 | RFWD3 | AGPAT5 | TIA1 | PPIE |
| TSGA10 | COA1 | MTF2 | NOB1 | ITPA | C2orf47 |
| TGOLN2 | ATP6AP1 | SLC25A40 | IVD | AP3M2 | COX15 |
| PECR | HIST1H2BJ | ZNF544 | POP1 | OAS1 | RFC3 |
| PPFIBP2 | TSFM | RBM41 | ANK3 | SPIRE2 | PDXK |
| PAXIP1 | ABHD17C | ROMO1 | AGER | RNF44 | SETD6 |
| GPRIN2 | GALNT14 | CTAGE5 | ARPC1B | SNRPA1 | DSN1 |
| PTPN4 | GGCX | ZNF91 | FBXO22 | ANKRD23 | MYO19 |
| SMC2 | CHML | MTBP | SLC25A15 | PPL | NSG1 |
| CADM1 | GMCL1 | ITGB3BP | IDE | MRPL37 | SETDB1 |
| CLNS1A | CCDC77 | COX5A | TREX1 | RRP1 | XPR1 |
| ALDH18A1 | SQLE | ALG8 | ZDHHC20 | POLD2 | ADPRHL1 |
| FBXL18 | C1orf35 | CSTF2 | CENPN | TIMM8A | LRRC6 |
| MRPS34 | SLC25A36 | MRPS25 | TSEN54 | YBX2 | PASK |
| HIST1H2BM | MAT1A | TEKT2 | DCAF11 | NFS1 | ISCA2 |
| ZNF253 | LGR4 | PTGS1 | ADCK5 | ARL6IP1 | PSME2 |
| TXNDC17 | ZNF101 | XPO1 | NADSYN1 | NDUFB3 | MRPL15 |
| ORC4 | BRCC3 | ACVR2B | SP1 | RASGEF1A | FANCG |
| BRI3BP | IMMP1L | PERP | STRC | UGGT1 | PIK3CB |
| RCC2 | GEMIN6 | RFC5 | SIRT5 | GOT1 | UNG |
| CCHCR1 | UMPS | AGBL5 | PGK1 | UBAP2 | CNNM4 |
| WDR90 | SDHAF4 | RIDA | CSE1L | TRIT1 | PTPN14 |
| PRELID3B | FAM134A | CNPY2 | TMEM254 | RIPK4 | MRPS26 |
| TMEM68 | PLAGL2 | CHEK2 | RMND5A | C20orf24 | PAICS |
| DDX49 | RCAN3 | DUS4L | NUDT5 | CKAP5 | AKR1A1 |
| FAM174B | TMEM141 | CYC1 | LZTFL1 | NHLRC3 | TSSC1 |
| ZNF664 | ZNF691 | TYSND1 | LRBA | DSCC1 | HIST1H2BL |
| 44629 | CD82 | EPPK1 | PDCD6 | GPR89B | FAM49B |
| CCP110 | PDCD2L | CYB561D2 | WDR73 |  |  |

| **down-regulated genes in GSE12470** | | | | | |
| --- | --- | --- | --- | --- | --- |
| FAM110D | CCSER2 | GPR146 | MAFK | RFX2 | COX4I2 |
| APOLD1 | GPRASP1 | TNFRSF1B | VEGFC | ANXA6 | DDX3X |
| SOCS2 | CD59 | PLAUR | VIM | MRAS | MAST4 |
| CLEC3B | CBX7 | F2R | CHSY1 | CCDC80 | ZDHHC14 |
| PI16 | LAMA4 | CRTC3 | GIMAP1 | BAG3 | PDGFA |
| ASB2 | MAF | LDLR | STAT5A | FGR | FAM167B |
| LGI4 | EPS8 | SLFN11 | NPDC1 | TMEM204 | S1PR4 |
| SPARCL1 | DCN | CEBPE | COL15A1 | LMCD1 | PPP1R14A |
| MGP | CDC42EP3 | MLLT11 | SERP2 | ZBTB4 | KANK1 |
| SGCA | BAMBI | NEDD9 | GMFG | SBDS | COL6A3 |
| TNXB | EMP1 | INPP4B | PPM1K | PRKCH | LOC338620 |
| KANK3 | SNRK | STAB1 | CHD1 | IER3 | DNAJC18 |
| DPT | SWAP70 | COL4A1 | KCTD20 | PRKCDBP | TTC7B |
| DES | MAOB | COL3A1 | ANGPT2 | SLC47A1 | MSC |
| EMCN | SORBS2 | PADI4 | MIDN | 44258 | DNAJB4 |
| CNN1 | SOBP | SATB1 | FOLR2 | SH3PXD2A | COL6A1 |
| CSRNP1 | ZNF106 | ADARB1 | AMOTL1 | TGFB3 | C10orf54 |
| DLC1 | HDAC4 | PLEKHA4 | PNPLA7 | GALNT18 | PPP3CC |
| KLF9 | MAP3K8 | CORO6 | KCTD10 | C1QTNF5 | AGPAT4 |
| RGS2 | CELF2 | MAPRE2 | ADAM19 | CPQ | SLC12A4 |
| PDE2A | STX2 | NRP2 | TUBE1 | CREM | GNG12 |
| KCNMB1 | MEF2C | SPSB1 | FCGRT | ID3 | CAMK2N1 |
| KLF2 | FCN1 | PRSS23 | APBB1 | PLXDC1 | CNKSR3 |
| RAMP2 | TIMP3 | LRMP | CTIF | MBNL1 | ZEB2 |
| AQP1 | FERMT2 | STARD5 | FAXDC2 | OLFML3 | TSPAN4 |
| HMCN2 | BMP2 | MGC24103 | ARSI | LTBP4 | KCNJ8 |
| NPY4R | ALDH1A1 | PLAGL1 | ABHD6 | COLGALT2 | LUM |
| ECSCR | FBXL7 | LRP1 | HCFC2 | RNF122 | CRIM1 |
| CNTNAP1 | STK26 | GPBAR1 | LATS2 | KLF6 | AKR1C3 |
| DACT3 | ANXA5 | WNT4 | VAMP5 | CLEC1A | GPER1 |
| ROBO4 | SAMD4A | VNN2 | ST6GALNAC6 | ASGR1 | PTRF |
| DSTN | GLIPR1 | AEBP1 | ROR2 | SPON2 | P3H3 |
| RAMP1 | SNCA | OSM | TFF3 | HBM | SORBS3 |
| SOX8 | IER5 | RASL12 | SFRP4 | MYL9 | MAPK12 |
| RGS5 | CAV2 | MYO15B | GOLIM4 | CSF3 | SULF2 |
| TPSAB1 | RABGAP1L | METRNL | ITGB1 | EMP3 | AP1S2 |
| AOC3 | SPG20 | HSPG2 | GIMAP4 | SNX9 | CCDC88A |
| RCAN1 | SMARCA2 | GLI2 | MOB2 | ALDH1B1 | CRY1 |
| NEXN | PIEZO2 | ZC4H2 | RNF115 | TSHZ3 | GPRASP2 |
| PECAM1 | TMEM47 | AHNAK | AES | MYOM1 | TNS2 |
| LMOD1 | ZBTB20 | CALHM2 | NPNT | DENND5A | PDLIM2 |
| CLDN5 | SDC2 | TMEM220 | PLK3 | NTRK2 | DDX5 |
| ZNF385B | TRAF5 | BASP1 | TMEM173 | SEPT10 | DUSP5 |
| GEM | TGFBR3 | ITIH5 | DYRK3 | C1orf54 | LOC102724428 |
| CLEC14A | DKK3 | MGLL | FRMD3 | COQ10B | MICAL1 |
| ZCCHC24 | FGL2 | SGCB | FOXC1 | MMP28 | HEPH |
| CCL2 | TSPAN5 | SLC6A2 | PHLDA1 | IER2 | GALNT16 |
| PKIG | NDN | ASIC1 | LILRB2 | SLC12A5 | MCL1 |
| IL3RA | FEZ2 | EFS | CSRP2 | JDP2 | ABL1 |
| JUNB | CFH | AKTIP | DOCK11 | TNS1 | MYH3 |
| LINC00341 | CAST | NOV | AAED1 | ABR | HPGDS |
| LIMS2 | DFNA5 | TMEM158 | ADAMTS1 | NLRP3 | MID1 |
| S1PR1 | CPE | MCF2L-AS1 | ANKRD50 | HLX | LHX6 |
| SOCS3 | GABARAPL1 | IRF1 | APC2 | RGS16 | PLEKHO1 |
| CSRP1 | ADH5 | FAM110B | CX3CL1 | SMOC2 | HTRA3 |
| ACVRL1 | KLF11 | COL5A2 | RCSD1 | BTG2 | ITPKB |
| BAG2 | PALMD | LZTS1 | SPOCD1 | SYNPO | ACKR3 |
| CDKN1A | PTGER4 | EFEMP2 | CPNE5 | FSTL3 | CYBRD1 |
| CXorf36 | TCEAL2 | IL1B | GNAZ | GLIPR2 | CYB5R3 |
| DNAJB5 | SGCE | ADCY5 | HES4 | SPATA3 | ANG |
| LHFP | RUNX1T1 | DUSP26 | SLC22A17 | LIMA1 | C15orf41 |
| WFDC1 | CAP2 | PNP | DMPK | MRVI1 | PRPH |
| KANK2 | GFPT2 | HOMER1 | GRAP | DYSF | THY1 |
| BDKRB2 | CALD1 | CFHR1 | MTRF1L | TMEM71 | FAP |
| RAB23 | AKAP13 | TNNC2 | FHL2 | IGFBP5 | ICAM1 |
| JAM2 | GPNMB | WNT10B | CCPG1 | CD248 | RUSC2 |
| GRASP | CIRBP | PALM | BAHCC1 | PLA2G4C | CNPY4 |
| TCEAL5 | NR3C2 | LPAR6 | PCOLCE | SOD3 | NBL1 |
| NFATC1 | MICAL2 | BGN | CD99 | CCL19 | CCL8 |
| C1QTNF2 | ARHGEF10 | GABBR1 | KCNK6 | CTGF | SPTBN4 |
| MCAM | SH3BGRL | LFNG | CFHR3 | ATP8B2 | LY96 |
| NR4A2 | EPB41L3 | IL17D | CCDC3 | SLC24A3 | SELM |
| GPR4 | G0S2 | PCDH7 | ZNF521 | IGFBP7 | WFS1 |
| ACTG2 | PLSCR4 | PLXNA2 | CYTH4 | LTBP1 | GUCY1A3 |
| NFIL3 | HEG1 | GPAT3 | RRAS | ICAM2 | PLA2G5 |
| GADD45B | LYST | SFXN3 | EID3 | TIPARP | PRX |
| COX7A1 | DAAM1 | FZD4 | MLKL | GYPC | OSBPL5 |
| NR4A1 | TCF4 | GAS6 | CNN3 | ARHGEF25 | GGTA1P |
| MASP1 | PPM1D | DPYSL3 | MYL4 | TMEM59L | SH3D19 |
| GPIHBP1 | RYBP | ELK3 | MAGEH1 | HSPB2 | AXL |
| FLNC | EFNB3 | KLF8 | CKB | GIMAP8 | ITGA11 |
| HBA2 | FEM1B | ID1 | B3GNT5 | CPXM2 | PCP4 |
| EPAS1 | ARMCX1 | CDA | GPSM1 | POU6F1 | SDC3 |
| POPDC2 | FAM46A | TES | LIN7B | C1QTNF5 | DLL1 |
| SERTAD1 | C1S | ZYX | FBN1 | MCF2L | C1R |
| CAB39L | SRGN | LOXL1 | KDELC1 | TMEM55A | PTH1R |
| MAFF | S100A8 | DZIP1 | ACOX2 | KLHL21 | CD99L2 |
| NOSTRIN | RBPMS | CSF3R | SPHK1 | HIC1 | DAB2 |
| FILIP1L | HSPA2 | KLHL5 | LGALS1 | CHRD | CHRNA10 |
| TPM2 | CLIP1 | PAPLN | KATNAL1 | PRRT2 | IL4R |
| SCHIP1 | PROS1 | TMEM2 | SYNGR1 | DTWD1 | NFIA |
| GJC2 | MAOA | COL12A1 | GPR183 | NFKB1 | LRRC8A |
| ZFP36 | SULF1 | NAALADL1 | LHFPL2 | SNAI1 | ADIRF |
| ITGA7 | BCL2 | LTBP2 | DLG4 | IGF1 | BEGAIN |
| HBA1 | EHBP1 | PPIC | CDIP1 | FRZB | LMNA |
| TSPYL2 | SNAI2 | ENTPD3 | ULK2 | PLVAP | SGK1 |
| GSTM5 | MEIS2 | CCDC149 | DAPK3 | CFL2 | GULP1 |
| CACNA1C | EML1 | PTHLH | MTMR9LP | NDRG2 | TNFAIP3 |
| ABLIM3 | SPRY1 | FLT3LG | TESC | TBX1 | NGFR |
| NRN1 | FGF13 | PTGDS | CAMK1 | ITGA6 | PTPRB |
| HABP4 | SACS | CKMT2 | EVL | HGF | CDC42EP2 |
| CCIN | OSTM1 | PIWIL4 | ERRFI1 | FMOD | ABCG2 |
| MAN1C1 | PCDH17 | FZD8 | SIK1 | ZNF516 | ITPR1 |
| TBXA2R | ALDH2 | TSHZ2 | P2RX2 | RND3 | STARD13 |
| A2M | PJA2 | INHBA | LAYN | ENPP2 | ELL2 |
| WISP2 | FAM13B | VCAN | DUSP8 | FOSL2 | MRGPRF |
| PIM1 | ARMCX3 | LYPLAL1 | CLIP3 | EHBP1L1 | NAV1 |
| VWF | GAS1 | SLC36A4 | LYL1 | ITGA1 | ADCY4 |
| C4orf32 | PER2 | MFAP5 | CBR3 | NPFF | MMP19 |
| OSR1 | EID1 | CPXM1 | MCHR1 | RGCC | TAGLN |
| MYADM | CDC42BPA | CNTN1 | CXCL3 | PLEKHO2 | ATP1B3 |
| HBEGF | ROCK1 | CORO2B | ROCK2 | EGFL7 | MATN2 |
| PLPP3 | OSBPL1A | TREM1 | DNAJB1 | FBXO30 | ACTA2 |
| NES | NR2F2 | HHEX | ACOT2 | TMEM200A | NFKBIZ |
| ITGB1BP2 | SEC23A | TGFB1 | LARGE1 | YPEL4 | EGR1 |
| MTURN | PBX3 | MORN5 | ISLR | MYC | PALLD |
| TCEAL1 | RAP2C | THBD | NUAK1 | COL6A2 | APOD |
| FBLN1 | TOR1AIP1 | CAV1 | FPR1 | TRPV2 | PHLDB2 |
| CYR61 | PDGFRL | FAM129A | RRAD | SERPINF1 | ANGPTL2 |
| MPRIP | SLIT2 | FRY | RGMA | FAM198B | CCDC107 |
| FBLN5 | ATP10D | SFRP1 | PPP6R1 | TBX3 | MFAP4 |
| CSPG4 | CCND2 | PLPP1 | SCARF2 | CXCL1 | TUBB6 |
| TMEM88 | METTL7A | STX12 | LCAT | ADGRA2 | ZBTB16 |
| PPP1R15A | FAM13A | CD44 | OLFML2B | SLIT3 | CRTAP |
| ERG | NCF2 | TFPI | RAB32 | EHD1 | MAPK11 |
| SYNC | NKX3-1 | IGFBP6 | TOM1L2 | TWIST1 | SMTN |
| GRK5 | PLS3 | KLF4 | BOC | ACTN1 | GSN |
| ITM2A | FAS | FHL1 | RAB11FIP5 | ENPEP | CRY2 |
| GJB3 | PPP1R12A | ETS2 | HVCN1 | SETBP1 | NOTCH4 |
| GJA4 | PCSK5 | FYCO1 | TRIB1 | EMILIN1 | ANTXR2 |
| ADAMTS5 | PTPRE | NAMPT | HSPA1A | MARVELD1 | FGF7 |
| PDLIM3 | HTRA1 | TPM1 | CDR2 | RIPK1 | JUND |
| CNRIP1 | DHRS7 | SYNE1 | DACT1 | KLHDC1 | DCHS1 |
| IGFBP4 | LAMB1 | HBB | THBS2 | ID2 | CXCL2 |
| TUBB2A | MTSS1 | TCEAL4 | MYO1C | NLRP1 | ADAM33 |
| LMO2 | HSD17B11 | CFD | LYNX1 | COL16A1 | PDE4B |
| SLC19A2 | SPTBN1 | GNG11 | ANO1 | GPR161 | MMP23B |
| WBP4 | TLE4 | PROCR | CCDC102B | SASH1 | CFP |
| GIMAP5 | SSPN | INPP1 | OR11A1 | DGKD | FOS |
| HEYL | SEPP1 | PDLIM5 | C1QTNF4 | CACNA1H | MAP3K7CL |
| PCDH12 | GATA6 | RECK | GSTA4 | JUN | PLPP7 |
| FLNA | PAPSS2 | NR2F1 | GLT8D2 | ITGA5 | RILPL2 |
| ANKRD35 | MNDA | FOXN3 | SEMA6A | VAT1 | SLCO2A1 |
| MMRN2 | PDPN | ARID5B | GP1BB | PDS5B | FBLN2 |
| SVIL | COL4A5 | PMP22 | RAB8B | HIGD1B | SLMAP |
| CCL21 | EPB41L2 | C7 | SNPH | CEBPD | F10 |
| NEFM | PTPN13 | TGFBR2 | PIK3R3 | BATF3 | IL34 |
| DCUN1D3 | NEK1 | KLF10 | EMILIN2 | COL4A2 | FEZ1 |
| SNX21 | PLCL2 | CSGALNACT1 | NNMT | CGNL1 | ILK |
| FSTL1 | LGALS2 | TACC1 | SPRYD3 | STBD1 | FAM65A |
| ZNF575 | NPTN | ITGA9 | BOK | PDLIM7 | FOSB |
| SH2D3C | AMIGO2 | BFSP1 | ARHGAP10 | REV3L | DUSP1 |
| MAP1A | FYN | CLEC2B | MT1M | DENND2A | CXCL12 |
| PRELP | GSTM3 | NLGN2 | MXRA8 | PHLDB1 | GADD45G |
| HBQ1 | PLA2G4A | SLC16A2 | BTG1 | LINC00597 | EGR2 |
| TEK | RAB31 | NOVA1 | COL1A2 | GDPD5 | PER1 |
| C16orf45 | FZD7 | CRAT | TLE2 | C1orf198 | TNC |
| ADGRF5 | RPL23AP32 | RAI14 | CD69 | KIAA0513 | RASSF2 |
| GIMAP6 | DPYD | PRKD1 | CRLF1 | NACAD | ATL1 |
| RASIP1 | SEMA3C | DUSP3 | PPP1R12C | CRYAB | STOM |
| RASD1 | BMP4 | PTPRM | NOS3 | ITPRIP | NSRP1 |
| ZNF331 | TF | FIBIN | AMFR | PCDH18 | PDGFRB |
| NFKBIA | PRNP | SYNJ2 | SPARC | CTSK | RBP7 |
| CPT1C | ACSL1 | FGFR1 | CACNB1 | ADCY3 | ESAM |
| LILRB3 | PTN | TGFBI | PKD1 | CASKIN2 | FXYD6 |
| SMAD7 | PDLIM4 | SLC43A1 | HOPX | ADGRE5 | LPP |
| PMEPA1 | INSIG1 | FABP3 | TSC22D2 | VENTX | KCTD17 |
| LSP1 |  |  |  |  |  |
