# Supplementary material for: Downregulated PRNP Facilitates Cell Proliferation and Invasion and Has Effect on the Immune Regulation in Ovarian Cancer
Source: J Immunol Res. 2022 Sep 29;2022:3205040. doi: 10.1155/2022/3205040 (PMC9537007; doi:10.1155/2022/3205040)
Supplement: Supplementary Materials — Supplementary Figure S1. The relationship between PRNP expression and the immunomodulators in ovarian cancer. (A) The relationship between PRNP expression and the immunostimulators. (B) The relationship between PRNP expression and the immunoinhibitors. Supplementary Figure S2. The relationship between PRNP expression and the chemokine and its receptors in ovarian cancer. (A) The relationship between PRNP expression and the chemokines. (B) The relationship between PRNP expression and the chemokine receptors. Supplementary Table S1. The upregulated and downregulated genes between normal ovary and ovarian cancer tissues from GSE12470. Supplementary Table S2. The upregulated and downregulated genes between normal ovary and ovarian cancer tissues from GSE26712. Supplementary Table S3. The ferroptosis-related genes. Supplementary Table S4. Logistic regression analysis of PRNP expression correlated with clinicopathological factors in ovarian cancer. [file 3205040.f1.zip › Supplementary Table S2..docx]

**Supplementary Table S2.** The up-regulated and down-regulated genes between normal ovary and ovarian cancer tissues from GSE26712.

| **up-regulated genes in GSE26712** | | | | | |
| --- | --- | --- | --- | --- | --- |
| FKBP8 | CCNB1 | SLC52A2 | IGFBP4 | PSMB3 | SRRM1 |
| SNORA68 | SLPI | FIBP | SEPW1 | JUN | FOXM1 |
| C9orf16 | KIF20A | MCM3 | IGK///IGKC | TSC22D2 | XAB2 |
| YIPF2 | SLC2A1 | NDUFB11 | PGGHG | COL18A1 | CDK16 |
| ATRIP | ELF3 | H2AFX | THBS1 | CHCHD2 | CFL1 |
| RPL37A | STXBP2 | PDCD5 | PTH2R | KLF6 | ARHGDIA |
| B3GAT3 | NUSAP1 | FBXL6 | PLPP3 | SPON1 | MCL1 |
| NOTCH3 | SLC39A4 | FKBP4 | CITED2 | SCAND1 | TCEB2 |
| PFN1 | ST14 | POLR2I | VCAN | CARM1 | H2BFS |
| CORO1B | PSAT1 | NDUFA7 | HSPA1B | TOP2A | BOLA2B |
| RNF19B | SPP1 | KRT23 | PGRMC1 | SOX12 | LRRC32 |
| OTUB1 | KLK6 | NR2F6 | ATP5J2 | GLDC | RHOB |
| RPS7 | ESPL1 | POR | CYAT1 | TAGLN | NUTF2P4 |
| MIR1282 | FOLR1 | TUBB | MEOX1 | SDHC | CALU |
| PABPN1 | ASF1B | UQCR10 | INHBA | BUB1 | MCM10 |
| ARPC4 | PRC1 | TACSTD2 | NUAK1 | L1CAM | EIF4G1 |
| PPP5C | ABHD11 | ATP6V0B | KLK7 | MLF2 | PKP4 |
| HIST2H2AA3 | CENPU | SNRPB | HSPB1 | SLC25A1 | FAM171A1 |
| PRKAR2A | CKS2 | TKT | TPM4 | ATP5I | DRAP1 |
| PRMT1 | APOC1 | S100A2 | GSN | BTG3 | EPHX1 |
| SCAMP4 | MAD2L1 | RPL39L | CNN3 | DLGAP5 | TAGLN2 |
| WFDC2 | SDC1 | PPP4C | GUCY1B3 | S100A4 | C20orf27 |
| PTMA | GRHL2 | ENO1 | IGFBP5 | TSC2 | BCL11A |
| CNOT3 | HIST1H2BD | MRPL4 | MMP2 | CALM3 | SF3A2 |
| ARF5 | RACGAP1 | MYO10 | INS-IGF2 | MFAP2 | FLOT1 |
| SOX17 | DTL | BZW2 | IGLV1-44 | COX6C | NEK2 |
| KLC2 | LSR | RPN1 | TFAP2A | REEP5 | CHIC2 |
| OGFR | AHCY | PRKCD | NPTX2 | MRPL42 | HNRNPL |
| MMP14 | PTPRF | COX6B1 | ACTG2 | MT1G | MIR6734 |
| AGPAT1 | BIK | PPDPF | MFAP5 | RHOC | SRM |
| FAXDC2 | CBS | ATP5G3 | IGHA2 | MT1HL1 | SETSIP |
| PEA15 | CCNE1 | AURKAIP1 | HNRNPC | LYPD1 | DGCR6L |
| CDS2 | ECT2 | TRIM27 | IGKV1OR2 | SNORD68 | CDC25A |
| PTK7 | TMPRSS4 | COL9A2 | IGLJ3 | SNCG | RALGDS |
| CLPTM1 | PAM16 | COPG1 | CLU | COX7C | ATN1 |
| ATP5H | TIMELESS | C19orf53 | PFDN4 | SNORA52 | OVOL2 |
| SRCAP | TACC3 | NTHL1 | IGLC1 | HSPD1 | PCDH7 |
| DBN1 | SORD | MT1F | MIR3620 | NCALD | RAB8A |
| ACTB | LSM4 | CKS1B | TXN | TNNT1 | LTBR |
| DDA1 | PSRC1 | CARHSP1 | PHLDA1 | FAM107A | TRIM28 |
| CRABP2 | UCP2 | ISG15 | LGALS1 | TGFB1 | FAM193B |
| HMGA1 | PDIA4 | MAL | IGKC | ATP6V1B1 | SLC44A4 |
| CHD8 | MUC1 | IRF9 | ISLR | SULT1C2 | LRRN2 |
| YWHAE | SFN | CHODL | HMGA2 | TWF1 | MFSD10 |
| INTS3 | MDK | AGRN | CCT2 | DNAH3 | ATP6V0C |
| TUBA1C | HDGF | DNPH1 | BMS1P20 | RPL10 | RAD23A |
| BCL7C | SCRIB | IFI27 | SDF4 | RPL36 | UBE2M |
| NUDT3 | MCM7 | SMG7 | APOA1 | SNRPD2 | LAMC1 |
| ANP32A | SHMT2 | S100A11 | IGHM | LRIG1 | BLOC1S1 |
| BTBD2 | NCBP2 | LAMTOR2 | COL6A3 | CEP55 | MIR4745 |
| ZNF593 | MIF | LSM8 | FOSB | CEBPG | PRKDC |
| H3F3AP4 | COX5B | NME1 | PTX3 | TUBB4B | TTK |
| MAP2K2 | RFXANK | HYOU1 | IGLJ3 | GPM6B | RPL36A |
| KDELR1 | GSTP1 | DCXR | SST | DDTL | ISOC2 |
| ERF | JUP | NDUFA13 | ZIC1 | PRAME | ZYX |
| DFFA | ABCF3 | HIST1H2BK | RAB25 | SLC34A2 | STT3A |
| NUCB1 | LAMP3 | NDUFA3 | MIR8071-2 | SSR4 | SLC27A3 |
| MAP7D1 | RNASEH2A | MSH6 | SELT | CLDN10 | JAG2 |
| HSPG2 | HIST1H1C | WBSCR22 | FBLN1 | CTNNB1 | FANCI |
| ZER1 | CDKN3 | PGLS | LGR5 | MT1H | YWHAH |
| HJURP | PRKCI | PCYT2 | MUC16 | ZWINT | PDE9A |
| C1orf186 | HIST1H2BH | IGFBP2 | COL5A1 | IFIT3 | HEY2 |
| ADAP1 | MRPL12 | RUVBL2 | CXCL14 | SERPINH1 | SIVA1 |
| TPX2 | LAPTM4B | MID1IP1 | MYL9 | LSM2 | SSB |
| HSF1 | EXOSC4 | UBE2S | SERPINE1 | CHST1 | TOMM22 |
| FASN | GPI | RBBP4 | MLIP | CEBPB | THOC6 |
| SNRPE | CDKN2A | UBAP2L | PTGDS | VDAC1 | HNRNPUL1 |
| NDUFS8 | PRSS2 | SYNGR2 | KRT7 | S100A14 | CHMP1B |
| PML | MRPS12 | SMC4 | GPX3 | DDAH2 | CCDC85B |
| UBE2Z | ASS1 | KLK5 | CTGF | DUSP5 | MIR7112 |
| SLC2A4RG | PRSS21 | PNN | FOS | CST3 | ESRP1 |
| LRP5 | CAPG | S100A13 | PKP3 | SLC6A8 | HOMER2 |
| HGS | ASRGL1 | LAD1 | SPINT2 | GADD45GIP1 | ERH |
| ADRM1 | PKM | BMP7 | EPCAM | YWHAZ | PLVAP |
| VWA1 | TSTA3 | BST2 | CLDN4 | SH3BGRL3 | JUNB |
| SMARCB1 | FARSA | PSMB2 | CLDN7 | P4HB | ERI3 |
| SLC25A11 | SCGB2A1 | LGALS3BP | PRSS8 | NEU1 | ZNF467 |
| PRAF2 | GAPDH | NUP62 | TK1 | SPINT1 | ZFP36L1 |
| C1orf106 | TPI1 | GCAT | UBE2C | COL6A1 | EMID1 |
| PTPRU | SMARCC1 | AKAP8L | CCNB2 | SAR1A | UBE2N |
| RAB5B | HN1 | TUBA4A | BIRC5 | IGKV1OR2 | SNORA3A |
| SDC3 | CHI3L1 | MMP7 | CENPF | COL15A1 | SUB1 |
| ARFIP2 | MRPL13 | IFI6 | BUB1B | TRIB2 | INHBC |
| COPE | IDH2 | CAPN1 | TRIP13 | LMNA | RALY |
| BANF1 | CALR | CD74 | DHCR24 | PLTP | SF3B4 |
| ACTN1 | ISYNA1 | H1FX | PAX8 | MIR6805 | SLC4A5 |
| ACTN4 | CCT5 | STIP1 | KIF2C | ATP6V0E1 | GINS1 |
| ARF6 | SORT1 | DCAF15 | CDCA8 | TRIM29 | AK4 |
| CENPT | PPIF | GCDH | CDK1 | FZD10 | IL32 |
| SNRNP40 | RAE1 | CXCR4 | CLDN3 | FZD2 | COL1A1 |
| S100A6 | PUF60 | VEGFA | KRT8 | VTCN1 | BRD2 |
| G6PD | PYCRL | MTHFD2 | AURKA | NDUFA4L2 | FUT8 |
| FKBP1A | DEFB1 | GSDMD | CDC20 | PBK | PKN1 |
| JUND | CD24 | PAK2 | MCM2 | RPL35 | PRPF4 |
| CDC42BPB | MECOM | MAZ | MCM4 | ITM2C | MT1X |
| CIC | HIST1H2BE | CXCL13 | FAM64A | LOXL1 | FXYD3 |
| TUBA1B | SCGB1D2 | NT5DC2 | TPD52 | IGLL3P | GPS1 |
| PRRC2A | LRFN4 | THEMIS2 | KIAA0101 | CAPNS1 | DGKZ |
| OAZ2 | EIF4EBP1 | AGPAT2 | PTTG1 | IGLL5 | UGT2B28 |
| SET | CYBA | KLHDC3 | KLK8 | ITGB4 | LZTS3 |
| EPHB2 | SLC50A1 | GRINA | TMED2 | HIST1H2BJ | NDUFB7 |
| PDLIM7 | MRPL2 | PRKCSH | NOP10 | PART1 | INO80B |
| UQCRQ | MARCKSL1 | RANGAP1 | EPB41L1 | COL6A2 | IGHG1 |
| TXN2 | PPP1CA | OASL | MCAM | CDH6 | PEBP1 |
| UPK3BL | RPN2 | CTSD | LAMP1 | SEC61G | SMTN |
| EIF5A | TRAF4 | TUBB3 | ATG101 | ZFP36L2 | LOXL2 |
| SETSIP | COX7B | RGS1 | BAG6 | COMP | RRBP1 |
| KIF4A | MPZL2 | APOE | ATOX1 | CBLC | RPL23 |
| CIZ1 | PPP1R14B | CRABP1 | HMMR | TNFRSF12A | S100A1 |
| RPL38 | ST6GALNAC2 | GANAB | DNAJB1 | CDK2AP2 | CRIP2 |
| OGDH | SOX9 | OXTR | C21orf33 | PRCC | CAPZB |
| CDC42EP4 | TECR | GPX4 | MMP11 | CSNK1A1 | POLR2J |
| ARID1A | BAK1 | TP53 | MARCKS | CCNA2 | PLD3 |
| COL4A1 | RBM38 | CP | BACE2 | PFKP | HIST1H2BG |
| GPBP1L1 | COX8A | BGN | RCN3 | CRTAC1 | ARPC4 |
| STAB1 | ERBB2 | TMEM97 | CKB | TYMP | KRIT1 |
| GALNT6 | ADGRG1 | THY1 | FOSL2 | MFGE8 | NDUFV2 |
| GDI1 | UCK2 | MCAM | INHBB | EVA1B | PTP4A3 |
| BCAM | FLNA | LSM7 | VWF | JUP | COL4A2 |
| GLB1L2 | LCN2 | TNFAIP2 | SLC16A3 | RGCC | COX6A1 |
| AAMP | ATP2A2 | TLN1 | UBA1 | HMGB1 | NES |

| **down-regulated genes in GSE26712** | | | | | |
| --- | --- | --- | --- | --- | --- |
| LGALS8 | TPM1 | CREB3L2 | WDR11 | KIAA0355 | GCA |
| BNC1 | SYNE1 | RANBP2 | NME7 | ICE2 | MICA |
| KDR | HBB | KIAA0232 | HPSE | TRA2A | PCCA |
| GPM6A | TCEAL4 | ASNSD1 | CHORDC1 | RUFY1 | RNF38 |
| NELL2 | CFD | UGP2 | LEPROT | STAG2 | HOXC6 |
| HNRNPD | GNG11 | PPP1CC | AVL9 | SEMA6D | UBXN2B |
| TMEM255A | PROCR | VAMP7 | GMFB | GRSF1 | SEC63 |
| FAM153B | INPP1 | ATP9A | CSF2RB | SLC25A44 | CRYBG3 |
| SLC4A4 | PDLIM5 | TLK1 | GOLGA8N | ANKRD10 | PITPNC1 |
| NPY1R | RECK | BECN1 | DRAM1 | REV1 | UBR2 |
| REEP1 | NR2F1 | IL13RA1 | PPIP5K2 | SIPA1L1 | TMED7 |
| PSD3 | FOXN3 | GNG10 | ALDH6A1 | SIK3 | WDFY3 |
| PRG4 | ARID5B | ANKRD36B | SRSF7 | ZNF83 | C1RL |
| CLDN15 | PMP22 | TDG | NAT1 | SLC16A5 | PNRC2 |
| CLEC4M | C7 | TJP1 | KYAT3 | ALDH1A3 | YTHDC2 |
| S100PBP | TGFBR2 | BEX4 | CLIC5 | TBC1D9 | ASPH |
| KIAA0226L | KLF10 | HIPK1 | RRAS2 | TASP1 | BICC1 |
| ANXA8L1 | CSGALNACT1 | SPCS2 | NAA16 | RBM26 | VAMP4 |
| WNT2B | TACC1 | CDKN1B | TNXB | CHMP5 | NANOG |
| SLC46A3 | CCSER2 | CCDC53 | NSA2 | WDR47 | ATXN1 |
| PTGDR | GPRASP1 | SQRDL | IL1R1 | NR3C1 | RAB1A |
| TFPI2 | CD59 | HEXB | MYRF | CAPN7 | MPHOSPH6 |
| LHX2 | CBX7 | AMOTL2 | UBA3 | PODXL | DDX17 |
| CYP39A1 | LAMA4 | JAK1 | MAX | LYVE1 | NR0B1 |
| MRPS14 | MAF | CKAP4 | APIP | CFHR1 | BCLAF1 |
| C21orf62 | EPS8 | MAP3K4 | RBM39 | TPBG | ANKRA2 |
| ZNF23 | DCN | PRRC2C | LY75 | SPTAN1 | ACPP |
| USP12 | CDC42EP3 | PI4KAP1 | ARL8B | RBM12 | CUX1 |
| PLCE1 | BAMBI | RPL15 | NEBL | TNFAIP8 | MPDZ |
| ATM | EMP1 | MIR4738 | EIF5 | FAM69A | SEL1L2 |
| ZNF45 | SNRK | MBD2 | CTSL | RCHY1 | CDC27 |
| CALB2 | SWAP70 | SEC14L1 | RCBTB1 | SLC7A8 | SH3GL2 |
| ZNF112 | MAOB | BNIP3L | SCP2 | ACACB | SVEP1 |
| DSC3 | SORBS2 | ITGAV | SYNE2 | SEH1L | ID2B///ID2 |
| MTUS1 | SOBP | NUP153 | TRO | TSPYL4 | SRSF10 |
| SPOCK1 | ZNF106 | CD53 | STK24 | GTF2H1 | EFHC1 |
| ZNF350 | HDAC4 | DNAJA1 | GATM | STXBP3 | GHR |
| PDE8B | MAP3K8 | LSM14A | ADD3 | DCP2 | MSRB2 |
| DCUN1D4 | CELF2 | AADAC | IFI16 | PTPRC | ECM2 |
| NFYB | STX2 | SETSIP | TSPAN8 | MDFIC | NEFH |
| MARK3 | MEF2C | GNAQ | ANAPC13 | NCOA1 | RAB27A |
| ARHGAP44 | FCN1 | ANP32B | HNRNPH3 | CDV3 | SETX |
| PCDH9 | TIMP3 | MRC1 | PCMT1 | FUBP3 | WWP2 |
| DPY19L1 | FERMT2 | CNOT2 | KIZ | ID4 | AGA |
| NBEA | BMP2 | SEC22B | SGMS1 | CLCN3 | UFL1 |
| ATP8A1 | ALDH1A1 | PLXNC1 | USP7 | ATP7A | MORC3 |
| RNF128 | FBXL7 | MFSD1 | RHOQ | SH3BP5 | LANCL1 |
| TCAF1 | STK26 | UNC50 | SLC30A9 | SFTPD | COL4A3BP |
| ADH1C | ANXA5 | RPA1 | UNC119B | ZBED5 | NT5E |
| AOX1 | SAMD4A | DYRK1A | NEK7 | DCLK1 | MEOX2 |
| SLC16A1 | GLIPR1 | LEPROT | HBA2 | ADCY9 | ZNF280D |
| PHLPP2 | SNCA | HNRNPA0 | TXNRD1 | HTATSF1 | DMD |
| PTGER3 | IER5 | HIBCH | IMPA1 | SF1 | TMX4 |
| CREBL2 | CAV2 | CBFB | FBXO28 | ABAT | TGFB2 |
| ALDH1A2 | RABGAP1L | MORF4L1 | CFDP1 | ECI2 | CREBZF |
| WNT5A | SPG20 | S100A10 | MAN1A1 | COLEC12 | RAB38 |
| BCHE | SMARCA2 | KDM2A | TMED10 | GRB10 | KIAA0430 |
| GCOM1 | PIEZO2 | DYNLT3 | SYBU | SHQ1 | EZR |
| RBL2 | TMEM47 | BRD8 | ZNF451 | RNMT | N4BP2L2 |
| SECISBP2 | ZBTB20 | MED21 | ITSN2 | BDH2 | PKN2 |
| FAM65B | SDC2 | HPR///HP | REEP5 | AKAP9 | HSPA12A |
| UHRF1BP1L | TRAF5 | PCM1 | RTN4 | DIAPH2 | RGL1 |
| PIP5K1B | TGFBR3 | ATG12 | ALG11 | OTUD4 | RABGAP1 |
| ME1 | DKK3 | 44811 | VPS13B | THAP9-AS1 | TBC1D4 |
| BTAF1 | FGL2 | CBX1 | NFIB | DNAJC8 | TBC1D12 |
| NRXN3 | TSPAN5 | ERLIN2 | MED4 | SFPQ | SERPINB9 |
| NAP1L3 | NDN | IPW | DPP8 | UBE3A | CHN2 |
| RTN1 | FEZ2 | KLHL2 | ATP6V1D | CAMSAP2 | BRD1 |
| IL18 | CFH | SNAP23 | ANXA4 | HS3ST1 | HNRNPDL |
| HSD17B2 | CAST | SLC30A1 | CAPN2 | PPP2R5C | DPP4 |
| AQP9 | DFNA5 | GOLGA8A | USO1 | FANCL | QKI |
| EP300 | CPE | AASDHPPT | FAT1 | ACAA2 | FAM208A |
| SGCG | GABARAPL1 | PDGFRA | KLC1 | CYB5A | RGS4 |
| TRPC1 | ADH5 | TANK | OARD1 | NTAN1 | CHUK |
| ZNF175 | KLF11 | DICER1 | SEC11A | GABARAPL1 | RNF219 |
| CSGALNACT2 | PALMD | SOD2 | RAP1A | AFF1 | THOC2 |
| TRAPPC11 | PTGER4 | COPS2 | SERINC1 | PLCB1 | WDR37 |
| ING3 | TCEAL2 | TRA2B | YME1L1 | PRKACB | NCBP3 |
| PCF11 | SGCE | CA12 | 44814 | RIOK3 | SEL1L3 |
| WSB1 | RUNX1T1 | EGFR | HACD1 | NLGN4X | DENND1B |
| DAB2 | CAP2 | FOXO3 | TM2D3 | KPNB1 | TRMT1L |
| ETFDH | GFPT2 | TM2D1 | MGEA5 | KIDINS220 | IPO5 |
| GLS | CALD1 | TMEM14A | ARID4B | SRP54 | FAM134B |
| SNCAIP | AKAP13 | CRYZ | RAB11A | SLC35A1 | PEX11A |
| CDC14B | GPNMB | GJA1 | PIKFYVE | MIS12 | PARVA |
| ST3GAL5 | CIRBP | HLA-E | SKIV2L2 | FBXO9 | IP6K2 |
| PTPRZ1 | NR3C2 | C5orf15 | GNS | RBFOX2 | RNASE4 |
| SNX1 | MICAL2 | HERC5 | SKAP2 | LUC7L3 | PSMC2 |
| SPAG9 | ARHGEF10 | MKL2 | NACC2 | ANKMY2 | PYGL |
| ZNF330 | SH3BGRL | ATG3 | KDM5B | CCNG2 | CASP7 |
| NAP1L2 | EPB41L3 | UAP1 | OSBPL9 | KRR1 | ATP2C1 |
| PHACTR2 | G0S2 | CCDC90B | AMMECR1 | NGLY1 | TMEM50A |
| RSRP1 | PLSCR4 | AP5M1 | ABCD3 | LRRFIP1 | SLITRK5 |
| ARAP2 | HEG1 | GHITM | SON | BLVRA | DIRAS3 |
| SLK | LYST | ZMYM2 | ARL6IP5 | PUM2 | SART3 |
| GSAP | DAAM1 | SMARCA5 | BTF3 | C2CD5 | IL16 |
| SLC31A2 | TCF4 | ARHGEF3 | CLK4 | ZCCHC8 | SYNJ1 |
| TMEM5 | PPM1D | TMEM123 | FBXL5 | SYPL1 | MIR22 |
| MTO1 | RYBP | ATP1B1 | HMGN4 | IL6ST | PKD2 |
| PPWD1 | EFNB3 | UQCRC2 | PYROXD1 | TXNDC15 | NSMAF |
| FRMD4B | FEM1B | FKBP1B | ZFYVE16 | LONP2 | OGN |
| SEMA5A | ARMCX1 | DEK | FAM120A | UPF3A | RUFY3 |
| RHOT1 | FAM46A | MIR5047 | RARRES1 | TERF1 | H6PD |
| FLRT2 | C1S | DENR | HNMT | USP34 | HBD |
| RYR2 | SRGN | CUL5 | RAB21 | VLDLR | PHF3 |
| CHGB | S100A8 | DERA | FOXO1 | ZNF226 | TSC1 |
| RDH14 | RBPMS | RFK | PNN | WT1 | GREB1 |
| VGLL3 | HSPA2 | RNF6 | RPL22 | CDK17 | FCGR3B |
| PAXBP1 | CLIP1 | TOP2B | FAM114A1 | SOS2 | TMEM9B |
| EPS15 | PROS1 | TAX1BP3 | RNF111 | RABGGTB | NDNF |
| PHKB | MAOA | GBP2 | CCDC47 | VPS4B | N4BP2L1 |
| CXorf57 | SULF1 | PPFIBP1 | HADHB | DCTD | CARMIL1 |
| MCTP2 | BCL2 | IFRD1 | GLUL | FRG1 | RBM5 |
| MOSPD2 | EHBP1 | MYH10 | BMI1 | GCC2 | ISOC1 |
| UFSP2 | SNAI2 | EDNRA | ENTPD4 | TIA1 | HAS1 |
| RAD17 | MEIS2 | NOTCH2NL | EMC2 | HEBP1 | IBTK |
| FAM134A | EML1 | PDCD6 | VSIG4 | ZCCHC10 | VPS13C |
| PLCG2 | SPRY1 | PPL | PSMA2 | ITM2B | RFC1 |
| EFEMP1 | FGF13 | CD46 | SERINC5 | NBPF20 | APPBP2 |
| TVP23B | SACS | SELENBP1 | DST | HSDL2 | ATP7B |
| GALNT12 | OSTM1 | SUCLG2 | PPP1R3C | TXNIP | HERC2 |
| MAP4K5 | PCDH17 | SMARCE1 | WAPL | ANGEL2 | WASF3 |
| AKAP7 | ALDH2 | CLIC4 | PID1 | CROCCP2 | OGT |
| ZDHHC17 | PJA2 | ADAM10 | YPEL5 | SCN3B | IQGAP1 |
| PCNX4 | FAM13B | CXADR | SRSF11 | DMXL1 | FOXJ3 |
| ITGAM | ARMCX3 | CCNC | ELOVL5 | C3AR1 | ATP9B |
| UBL3 | GAS1 | SYNCRIP | ZFAND6 | ZNF277 | SLC2A5 |
| PEX12 | PER2 | CD164 | SUCO | UBE2J1 | TTC37 |
| CSTF2T | EID1 | ZEB1 | GOSR1 | PLIN2 | THUMPD1 |
| XPA | CDC42BPA | CTNNAL1 | RDX | MS4A4A | CMAHP |
| ATF7IP2 | ROCK1 | SPTLC1 | GGNBP2 | GOLM1 | CASD1 |
| ERCC5 | OSBPL1A | HLA-DQB1 | PIN4 | GATA4 | CHMP3 |
| ANKRD12 | NR2F2 | ESD | CDCP1 | IRS1 | TNKS |
| CHRDL1 | SEC23A | HOXD4 | DHX15 | MCUB | METTL3 |
| TRIM2 | PBX3 | YTHDF3 | ST13 | UPK1B | MAP3K5 |
| PCOLCE2 | RAP2C | UBQLN2 | HP | ZFPM2 | TARDBP |
| APPL1 | TOR1AIP1 | F13A1 | CRNKL1 | LTA4H | FAM179B |
| CADPS2 | PDGFRL | PDE1A | SMCO4 | CYP2B6 | SNX7 |
| ARMC8 | SLIT2 | C11orf58 | SH3BP4 | IKBKB | SIRPA |
| AKT3 | ATP10D | HNRNPM | MS4A6A | HSD17B4 | WDR19 |
| CCDC93 | CCND2 | EMC7 | SRSF1 | ADRA2A | KCTD7 |
| ATMIN | METTL7A | ODC1 | CSTA | PTGIS | ADGRG6 |
| BCAR3 | FAM13A | USP33 | GLT8D1 | INSR | CCDC28A |
| OLFML1 | NCF2 | KLK11 | WLS | KCNJ2 | CCNT2 |
| DPYS | NKX3-1 | C3 | AAMDC | ASAH1 | GSPT2 |
| TNPO1 | PLS3 | C4B_2 | PEG3 | CTDSPL | STXBP1 |
| MARCO | FAS | CNIH1 | SLCO3A1 | TOB1 | DIXDC1 |
| MET | PPP1R12A | SERPING1 | LEPROTL1 | MUT | PIAS1 |
| SCTR | PCSK5 | CD163 | MEF2A | FKBP11 | RASA1 |
| NOL7 | PTPRE | TNS3 | NFE2L2 | SDCBP | CFI |
| DSE | HTRA1 | OSBPL8 | CXCL6 | CHD9 | FBXO38 |
| COBLL1 | DHRS7 | SNORD23 | RWDD1 | UBE2G1 | HECTD4 |
| TRIM68 | LAMB1 | ALG5 | 44626 | SAR1A | UBE2B |
| PGRMC2 | MTSS1 | LAMTOR3 | CTSO | CETN3 | C4BPA |
| AGGF1 | HSD17B11 | CTSC | MAT2B | RCBTB2 | SLC35G2 |
| IFFO1 | SPTBN1 | TMEM100 | NOC3L | ATP5L | TNFRSF10B |
| DAPK1 | TLE4 | ANXA1 | AHSA2 | OAT | CHST15 |
| DNAJB9 | SSPN | PSMC6 | TUBGCP3 | CERK | ARFIP1 |
| CUL3 | SEPP1 | EFR3A | P4HA2 | RAB22A | PURA |
| ZNF37BP | GATA6 | SNORD14D | TDP2 | SYNE3 | ANOS1 |
| GIPC2 | PAPSS2 | SMARCD3 | RPS6KA2 | RAP2B | EIF4B |
| ZKSCAN7 | MNDA | VCAM1 | MYCBP2 | PIGK | MED13 |
| HSD17B6 | PDPN | TMEM45A | PCDHA1 | SPG11 | SLC30A5 |
| PREPL | COL4A5 | MICU2 | NQO1 | C1GALT1C1 | DHX29 |
| PRDM10 | EPB41L2 | CCNG1 | DAZAP2 | PPFIA1 | NSG1 |
| MED14 | PTPN13 | PPP2CB | FNBP4 | NDUFA5 | RBM25 |
| TTC28 | NEK1 | MMADHC | HEXIM1 | SMAD2 | PICALM |
| VPS54 | PLCL2 | BNIP3 | MAP4 | BNC2 | HLF |
| SAP18 | LGALS2 | PEX2 | TMEM165 | ABI1 | GRAMD1C |
| TAOK3 | NPTN | GCH1 | LZTFL1 | ROBO1 | ACOT9 |
| ADO | AMIGO2 | TRIM22 | SLC39A6 | CYFIP1 | ATF1 |
| NEK9 | FYN | 44819 | SIAH1 | TRIM33 | SH3GLB1 |
| ALAD | GSTM3 | KRT19 | PNMA1 | LIPA | KIF13B |
| PIGB | PLA2G4A | XIST | STAM | HECA | HERC2P9 |
| CACNB2 | RAB31 | FBXO21 | TMOD1 | CBR4 | TCF21 |
| HERC1 | FZD7 | SNURF | MACF1 | ROR1 | CPVL |
| SNORD45C | RPL23AP32 | HSPA1B | TGOLN2 | RB1 | SARAF |
| ZNF573 | DPYD | BEX1 | EIF1B | SUN1 | ANXA3 |
| GCNT1 | SEMA3C | LYZ | LAMP2 | AGL | NBR1 |
| HAND2-AS1 | BMP4 | SPOCK2 | MIR4680 | ZNF395 | CAND2 |
| GALC | SLC39A8 | HAT1 | AUH | RUBCN | IVNS1ABP |
| PNISR | ACSL3 | CD14 | BTN3A2 | KDM6A | EVI5 |
| DZIP3 | ATG5 | ABLIM1 | UPK3B | SEC24B | MFF |
| PDGFD | NCOA4 | FAM3C | ZFAND1 | BTN3A3 | BCL2L13 |
| HS2ST1 | CYBB | LOC100272216 | NMRK1 | BIRC3 | UBE4B |
| ARHGAP6 | SAT1 | CAPZA2 | KIAA0485 | PIK3R4 | PROSC |
| WDR44 | PRNP | PSMB9 | GALNT1 | ATP6V1A | BBOF1 |
| WRB | ACSL1 | CCNI | TOM1L1 | SNX3 | TUSC3 |
| CLK1 | TBC1D2B | CD55 | ARMT1 | EVI2B | RBPJ |
| FGF1 | LPAR1 | PLOD2 | CX3CR1 | CAB39 | KIAA1109 |
| ADAMTS3 | PAFAH1B1 | CDH11 | TTC3P1 | SRSF5 | SHTN1 |
| CASP1 | PRKAR2B | EMX2 | PRKD3 | MDC1 | TMEM168 |
| LUC7L2 | LOC728392 | STAR | LGALS3 | AKAP17A | FBXW11 |
| RAB11FIP2 | TJP2 | ACTG1P4 | RPL31 | SPAG16 | DCAF6 |
| KAT2B | SCG5 | THBD | H2AFV | KDSR | RPL37A |
| GPR137B | ANK3 | CAV1 | CREBBP | UTP3 | FLRT3 |
| HBG2 | MED13L | FAM129A | RAN | PNMAL1 | HMGCR |
| UBE2I | BPTF | FRY | WTAP | FBP1 | PER3 |
| ALDH3A2 | LXN | SFRP1 | MCFD2 | SMARCA1 | PDE4DIP |
| NF1P9 | DMXL2 | PLPP1 | SPATS2L | YLPM1 | SACM1L |
| ABCA8 | TIAM1 | STX12 | IKBKAP | RAPGEF2 | CYP3A5 |
| LIN7C | ATP11B | CD44 | AGTR1 | LRRC1 | FGF9 |
| MST1 | ZBTB1 | TFPI | INPP5F | RALGAPA1 | SLC25A36 |
| 44628 | MST1 | IGFBP6 | EPB41L4B | DDHD2 | PTPRO |
| TRAPPC10 | PIK3R1 | KLF4 | TPP1 | CHMP2B | SLC38A6 |
| SCAMP1 | IK | FHL1 | PDZD8 | LIMCH1 | GNAI1 |
| TPD52L1 | FNDC3A | ETS2 | ATP2B1 | DPYSL2 | ATRX |
| SLTM | TSPYL1 | FYCO1 | CTNND1 | FAM8A1 | NPEPPS |
| AKAP11 | DOCK4 | NAMPT | FZD1 | NAV3 | PTCH1 |
| PDZD2 | DPY19L2P2 | DKFZP586I1420 | CRIM1 | DLG5 | ALDH9A1 |
| ITPR2 | USP3 | HNRNPA3 | PRKCA | BACH1 | GALNT11 |
| INSIG2 | DNAJC16 | ZNF302 | PDGFC | ZBTB18 | TBP |
| PTP4A2 | KIAA0368 | FBXO3 | NUDT9 | VWA8 | FBXW2 |
| ADAM9 | KHNYN | ADH1B | PIK3C3 | MAGI2 | MBP |
| PRKAR1A | RDH11 | C6orf120 | ATXN7 | COX20 | TRAK2 |
| ANKHD1 | TSPAN13 | ASH2L | PPP3CB | ISCA1 |  |
