# Supplementary material for: Downregulated PRNP Facilitates Cell Proliferation and Invasion and Has Effect on the Immune Regulation in Ovarian Cancer
Source: J Immunol Res. 2022 Sep 29;2022:3205040. doi: 10.1155/2022/3205040 (PMC9537007; doi:10.1155/2022/3205040)
Supplement: Supplementary Materials — Supplementary Figure S1. The relationship between PRNP expression and the immunomodulators in ovarian cancer. (A) The relationship between PRNP expression and the immunostimulators. (B) The relationship between PRNP expression and the immunoinhibitors. Supplementary Figure S2. The relationship between PRNP expression and the chemokine and its receptors in ovarian cancer. (A) The relationship between PRNP expression and the chemokines. (B) The relationship between PRNP expression and the chemokine receptors. Supplementary Table S1. The upregulated and downregulated genes between normal ovary and ovarian cancer tissues from GSE12470. Supplementary Table S2. The upregulated and downregulated genes between normal ovary and ovarian cancer tissues from GSE26712. Supplementary Table S3. The ferroptosis-related genes. Supplementary Table S4. Logistic regression analysis of PRNP expression correlated with clinicopathological factors in ovarian cancer. [file 3205040.f1.zip › Supplementary Table S4.docx]

| Characteristics | Total(N) | Odds Ratio(OR) | P value |
| --- | --- | --- | --- |
| Age (>60 vs. <=60) | 379 | 0.555 (0.368-0.834) | 0.005 |
| Race (White vs. Asian) | 340 | 0.750 (0.218-2.397) | 0.629 |
| Histologic grade (G3&G4 vs. G1&G2) | 369 | 0.900 (0.482-1.672) | 0.738 |
| FIGO stage (III&IV vs. I&II) | 376 | 1.432 (0.624-3.404) | 0.401 |
| Primary therapy outcome (SD vs. CR) | 238 | 3.036 (1.199-8.731) | 0.026 |
| Venous invasion (Yes vs. No) | 105 | 1.158 (0.528-2.554) | 0.714 |
| Lymphatic invasion (Yes vs. No) | 149 | 0.833 (0.417-1.658) | 0.603 |
| Tumor status (With tumor vs. Tumor free) | 337 | 1.379 (0.818-2.339) | 0.229 |

Supplementary Table S4. Logistic regression analysis of PRNP expression correlated with clinicopathological factors in ovarian cancer.
